# Xylosylated Detoxification of the Rice Flavonoid Phytoalexin Sakuranetin by the Rice Sheath Blight Fungus *Rhizoctonia solani*

**DOI:** 10.3390/molecules23020276

**Published:** 2018-01-29

**Authors:** Shun Katsumata, Hiroaki Toshima, Morifumi Hasegawa

**Affiliations:** College of Agriculture, Ibaraki University, 3-21-1 Chuo, Ami, Ibaraki 300-0393, Japan; 16am202l@vc.ibaraki.ac.jp (S.K.); hiroaki.toshima.spb540@vc.ibaraki.ac.jp (H.T.)

**Keywords:** phytoalexin, flavonoid, rice, *Rhizoctonia solani*, xylosylation

## Abstract

Sakuranetin (**1**) is a rice flavanone-type phytoalexin. We have already reported that the metabolites from the detoxification of **1** by *Pyricularia* *oryzae* are naringenin (**2**) and sternbin. In this study, we investigated whether the rice sheath blight fungus *Rhizoctonia* *solani*, another major rice pathogen, can detoxify **1**. The extract of *R*. *solani* suspension culture containing **1** was analyzed by LC-MS to identify the metabolites of **1**. Three putative metabolites of **1** were detected in the extract from the *R*. *solani* suspension culture 12 h after the addition of **1**, and they were identified as **2**, sakuranetin-4′-*O*-β-d-xylopyranoside (**3**), and naringenin-7-*O*-β-d-xylopyranoside (**4**) by NMR, LC-MS/MS, and GC-MS analyses. The accumulation of **2**, **3**, and **4** reached their maximum levels 9–12 h after the addition of **1**, whereas the content of **1** decreased to almost zero within 9 h. The antifungal activities of **3** and **4** against *R*. *solani* were negligible, and **2** showed weaker antifungal activity than **1**. We concluded that **2**, **3**, and **4** are metabolites from the detoxification of **1** by *R*. *solani*. Xylosylation is a rare and efficient detoxification method for phytoalexins.

## 1. Introduction

Phytoalexins are antimicrobial secondary metabolites that are produced in plants de novo after pathogen attack [1]. In rice plants, 19 phytoalexins have been reported, including 14 labdane-related diterpenes (momilactones, oryzalexins, and phytocassanes), one casbene-type diterpene (*ent*-10-oxodepressin), one flavanone (sakuranetin), and two amides (*N*-benzoyltryptamine and *N*-cinnamoyltryptamine) [2]. We demonstrated that the rice phytoalexins play an important role in blast disease resistance in rice plants [3,4,5].

Phytopathogenic microorganisms can detoxify phytoalexins to prevent their antimicrobial activities [6,7,8,9]. We have previously reported that the rice diterpenoid phytoalexin momilacotone A and the flavonoid phytoalexin sakuranetin (**1**) can be metabolized and detoxified by the rice blast fungus *Pyricularia oryzae* [3,5,10,11]. The detoxified metabolites of **1** were identified as naringenin (**2**) and sternbin, which are derived from **1** via 7-*O*-demethylation and 3′-hydroxylation, respectively [10].

**Figure 1:**
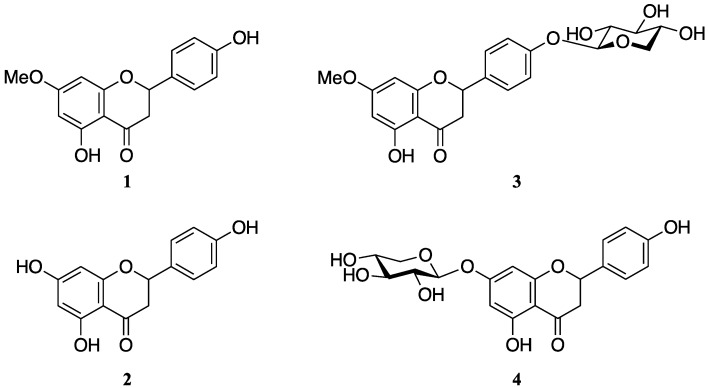
Structures of sakuranetin (**1**), naringenin (**2**), sakuranetin-4′-*O*-β-d-xylopyranoside (**3**), and naringenin-7-*O*-β-d-xylopyranoside (**4**).

Rice sheath blight fungus, *Rhizoctonia solani*, is another major fungal pathogen in rice [12], and **1** has been reported to show antifungal activity against *R*. *solani* [13]. We are interested in whether *R*. *solani* can also detoxify **1** and whether the detoxified metabolites are different from those of *P*. *oryzae*. In this study, the detoxified metabolites of **1** were identified from the *R*. *solani* suspension culture. We identified two xylosylated flavanones specific to *R*. *solani*, sakuranetin-4′-*O*-β-d-xylopyranoside (**3**) and naringenin-7-*O*-β-d-xylopyranoside (**4**), as well as **2**, which is also a metabolite of *P*. *oryzae*, as the detoxified metabolites of **1** (Figure 1).

## 2. Results and Discussion

### 2.1. Sakuranetin *(**1**)* is Metabolized by Rhizoctonia solani

**Figure 2:**
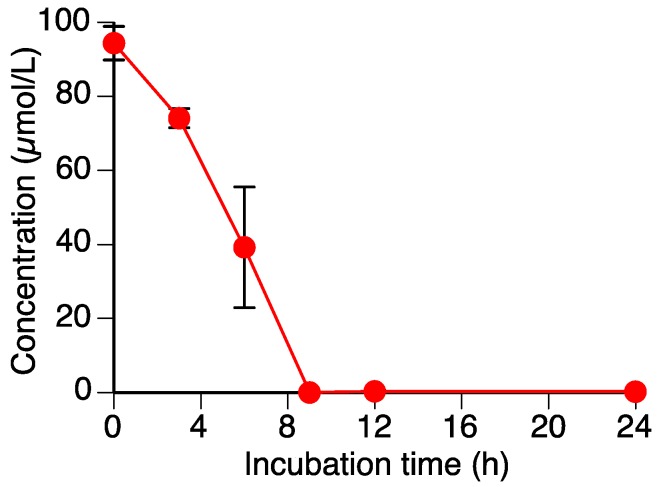
Time-dependent decrease in sakuranetin (**1**) content in the *Rhizoctonia solani* suspension culture. Values are reported as the mean ± SD (*n* = 3).

As shown in Figure 2, the level of sakuranetin (**1**) decreased to almost zero within 9 h in the *R*. *solani* suspension culture. We have already shown that another rice pathogenic fungus, *P*. *oryzae*, can metabolize **1**, which suggested that *R*. *solani* could also metabolize **1**.

**Figure 3:**
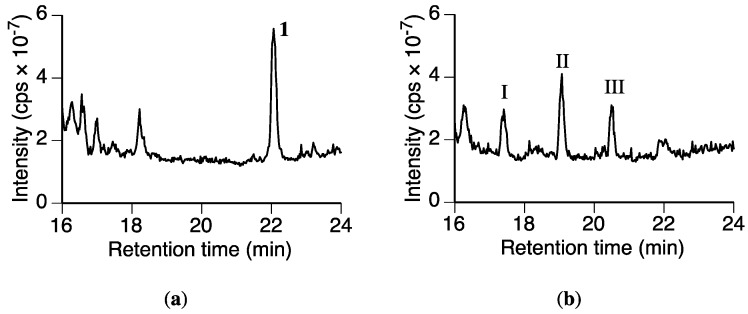
Total ion current chromatograms obtained from the *Rhizoctonia solani* suspension cultures containing sakuranetin (**1**) using LC-MS. (**a**) Chromatogram after 0 h of incubation following the addition of **1**; (**b**) 12 h of incubation after the addition of **1**.

We then screened the fungal culture for putative metabolites of **1**. The MeOH extracts from the fungal culture containing **1** after incubation for 0 and 12 h were analyzed by LC-MS. The total ion current chromatograms of each extract were compared (Figure 3). Compound **1** was detected in the 0 h extract at *t*_R_ 22.1 min, whereas only a trace amount of **1** was present in the 12 h extract. Three new peaks were detected at *t*_R_ 17.4 (I), 19.1 (II), and 20.5 min (III) in the 12 h extract. These peaks were not detected in a culture without **1** or in the medium containing **1** without the fungus (Figure S1). Therefore, we were certain these peaks were from the possible metabolites of **1**.

### 2.2. Identification of Naringenin *(**2**)* in the Rhizoctonia solani Suspension Culture Containing Sakuranetin *(**1**)*

**Figure 4:**
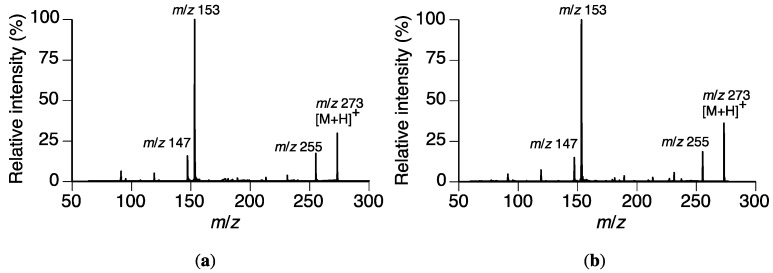
ESI-CID-MS results of a potential metabolite (peak II) in the *Rhizoctonia solani* suspension culture containing sakuranetin (**1**) and naringenin (**2**). (**a**) Peak II; (**b**) naringenin (**2**).

The mass spectrum of peak II showed a plausible [M + H]^+^ ion at *m*/*z* 273, which was consistent with the [M + H]^+^ of naringenin (**2**). The *t*_R_ of II (19.1 min) coincided that of **2**, which had been identified as a metabolite of **1** by *P*. *oryzae* in our previous study [10]. The *t*_R_ and collision-induced dissociation-mass spectrum (CID-MS) of peak II were compared with those of **2** in LC-MS/MS analysis. The *t*_R_ and CID-MS were consistent as shown in Figure 4. Therefore, we concluded that peak II was from **2**.

### 2.3. Purification and Identification of Sakuranetin-4′-O-β-d-xylopyranoside *(**3**)* and Naringenin-7-O-β-d-xylopyranoside *(**4**)* in Rhizoctonia solani Suspension Culture Containing Sakuranetin *(**1**)*

Peaks I and III showed significant ions at *m*/*z* 273 and 287, respectively, in their electrospray ionization-mass spectra (ESI-MS) (Figure S2). The ions are identical to the protonated molecules of **2** and **1**, respectively; however, their *t*_R_s are different from those of **2** and **1**. Therefore, we speculated that peaks I and III are derived from compounds that include naringenin and sakuranetin moieties, respectively.

We then tried to purify compounds **3** (peak III) and **4** (peak II) from the *R*. *solani* suspension culture containing **1** based on LC-MS analysis. Compounds **3** (1.1 mg, a white solid) and **4** (2.4 mg, a yellow oil) were successfully purified from 300 mL of the *R*. *solani* suspension culture containing 10 mg of **1**. Finally, 4.4 mg of **3** and 9.4 mg of **4** were obtained from 50 mg of **1** after several purification steps.

**Figure 5:**
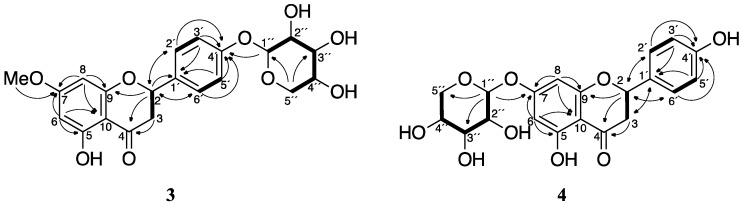
Key 2D NMR correlations for compounds **3** and **4**. The COSY and TOCSY correlations are represented by bold lines, and the HMBC are represented by arrows from H to C.

The ESI-MS of **3** showed plausible [M + H]^+^ and [M + Na]^+^ ions at *m*/*z* 419 and 441, respectively, suggesting a molecular weight of 418 (Figure S2). High-resolution mass spectrometry (HRMS) analysis using fast atom bombardment (FAB) ionization of **3** suggested a molecular formula of C_21_H_22_O_9_, which has eleven degrees of unsaturation. The ^13^C NMR and DEPT spectra revealed that **3** has 19 inequivalent carbons; namely, one methyl, two methylenes, nine methines, and seven quaternary carbons (Table 1). The ^1^H, ^13^C, HSQC, COSY, and HMBC spectra suggested the structure of sakuranetin is conserved in **3** (Table 1 and Figure 5). The TOCSY and COSY correlations of the remaining signals and their chemical shifts indicated that a pentose moiety should be present in **3**. The HMBC data suggested that the pentosyl group forms a pentopyranosyl structure, and that the pentopyranosyl group is connected to the oxygen at the 4′-position in sakuranetin.

The pentopyranosyl group in **3** was determined by GC-MS analysis of the hydrolysate of **3**. The hydrolysate of **3** was trimethylsilylated and then subjected to GC-MS. d-Xylose, d-ribose, and l-arabinose, which are common pentoses in natural products, were also trimethylsilylated and analyzed by GC-MS. The chromatograms of the trimethylsilylated (TMS) derivatives of the pentoses showed four peaks, which were indicative of the α-pyranose, β-pyranose, α-furanose, and β-furanose forms of the compounds [14]. The TMS derivative of the hydrolysate of **3** showed four peaks with *t*_R_s that coincided with those of xylose. The mass spectra of the four peaks derived from the hydrolysate of **3** also coincided with those of the xylose derivatives (Figure S3). Although the absolute stereochemistry of xylose was not determined experimentally, the d-form is a reasonable assignment because the l-form is not known as a natural product. The anomeric position was determined to be in the β-configuration based on the *J* value (7.2 Hz) of H-1″. We therefore concluded that **3** is sakuranetin-4′-*O*-β-d-xylopyranoside (Figure 1). The stereochemistry of C-2 was not determined in this study.

The ESI-MS of **4** showed a plausible [M + Na]^+^ ion at *m*/*z* 427, suggesting a molecular weight of 404 (Figure S2). HRMS analysis using FAB ionization of **4** was indicative of a molecular formula of C_20_H_20_O_9_, which includes eleven degrees of unsaturation. Some of the ^13^C NMR signals of **4** were observed as double signals that had very similar, but not identical, chemical shifts (Table 1). This suggested that purified **4** was a mixture of two diastereomers. However, the diastereoisomers were not chromatographically separable. We determined **4** was a diastereomeric mixture by a similar manner as we used to determine the structure of **3**. The NMR spectra of **4** revealed that a pentopyranose was connected to the 7-oxygen in naringenin (Table 1 and Figure 5). GC-MS analysis revealed that the pentose is a xylose moiety (Figure S4). We thus concluded that **4** is naringenin-7-*O*-β-d-xylopyranoside (Figure 1). The difference in the diastereomers must be the stereochemistry of C-2. However, the NMR spectra of **3** indicated that purified **3** seemed to be the single diastereomer. This suggested that *R*. *solani* may preferentially xylosylate a specific enantiomer of **1**.

Naringenin-7-*O*-xyloside was reported as a biotransformation product of naringenin by genetically engineered *Escherichia coli* that could express a glycosyltransferase from *Arabidopsis thaliana* and some other related enzymes [15]. The structure of naringenin-7-*O*-xyloside was confirmed only by LC-MS/MS analysis in that report.

### 2.4. Accumulation of Naringenin *(**2**)*, Sakuranetin-4′-O-β-d-xylopyranoside *(**3**)*, and Naringenin-7-O-β-d-xylopyranoside *(**4**)* in the Rhizoctonia solani Suspension Culture Containing Sakuranetin *(**1**)*

**Figure 6:**
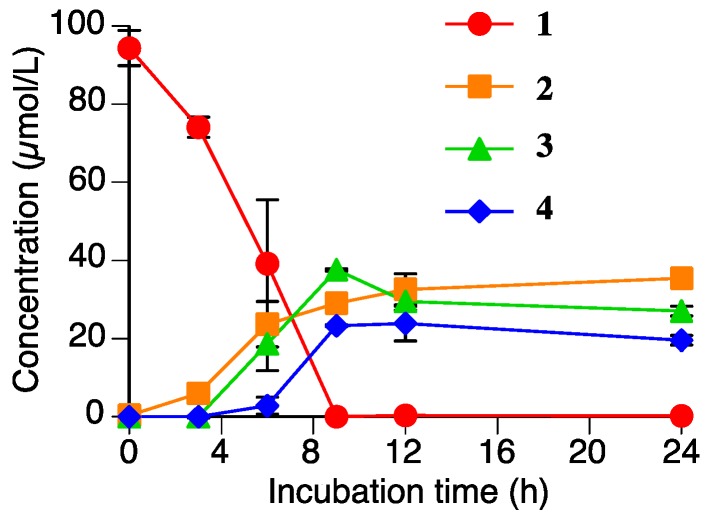
Time-dependent accumulations of naringenin (**2**), sakuranetin-4′-*O*-β-d-xylopyranoside (**3**), and naringenin-7-*O*-β-d-xylopyranoside (**4**), and the decreasing sakuranetin (**1**) content in the *Rhizoctonia solani* suspension culture. Values are presented as the mean ± SD (*n* = 3).

Figure 6 shows the time-dependent accumulation of **2**, **3**, and **4** in the *R*. *solani* suspension culture containing **1** (100 µmol/L). The level of **1** decreased to nearly zero in the 9 h after the addition of **1**. The levels of **2**, **3**, and **4** gradually increased in the first 12 h, and the levels were relatively constant until 24 h. This result strongly supported that **2**, **3**, and **4** are products of the metabolism of **1** by *R*. *solani*. The total accumulation of **2**, **3**, and **4** in the suspension culture after 9 h of incubation was 90 µmol/L. Therefore, these three compounds must be the major metabolites of **1** in the *R*. *solani* suspension culture.

We have already reported that **1** can be metabolized by *P*. *oryzae*, and that the metabolites were identified as **2** and sternbin [10]. Compound **2** is a common metabolite to both *P*. *oryzae* and *R*. *solani*, but the xylosylated flavanones, **3** and **4**, are specific to *R*. *solani*. Our previous study demonstrated that the accumulation levels of the metabolites of *P*. *oryzae*, **2** and sternbin, reached their maximum levels after 6–8 h of incubation and then decreased to the almost zero as the incubation time increase to 24 h [10]. However, the levels of the metabolites from *R*. *solani*, **2**, **3**, and **4** did not decrease significantly in 24 h of incubation.

### 2.5. Antifungal Activities of Naringenin *(**2**)*, Sakuranetin-4′-O-β-d-xylopyranoside *(**3**)*, and Naringenin-7-O-β-d-xylopyranoside *(**4**)*

**Figure 7:**
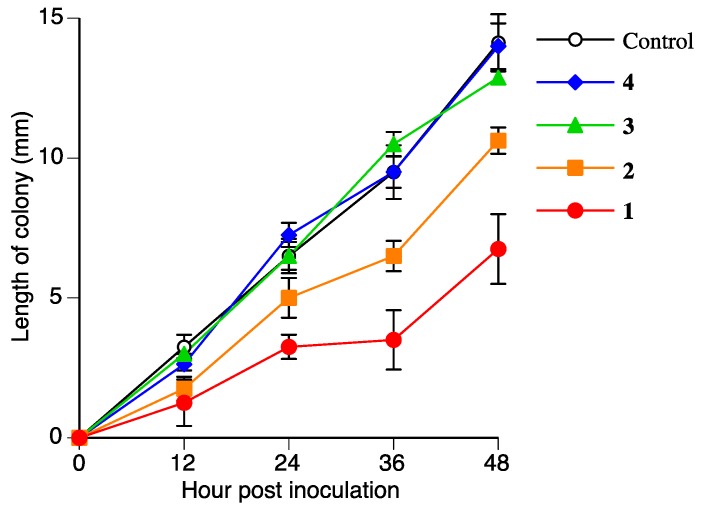
Antifungal activities of **1**, **2**, **3**, and **4** (300 µmol/L) against *Rhizoctonia solani* mycelium growth. Values are presented as the mean ± SD (*n* = 4).

The antifungal activities of **2**, **3**, and **4** were measured to determine if **2**, **3**, and **4** are detoxified metabolites of **1**. Figure 7 and Table 2 show the antifungal activities of **2**, **3**, and **4** against *R*. *solani*. The antifungal activity of **2** was lower than that of **1**. The antifungal activities of **3** and **4** were almost negligible. This result suggested that *R*. *solani* could detoxify **1** in the suspension culture.

Glucosylation is known to be a common detoxification method for phytoalexins [6,9]. However, to the best of our knowledge, xylosylation detoxification of a phytoalexin has not been reported. We therefore concluded that xylosylation is a rare and efficient mode of detoxification of phytoalexins.

## 3. Materials and Methods

### 3.1. General Analytical Methods

^1^H NMR (400 MHz), ^13^C NMR (100 MHz), and 2D-NMR spectra were acquired on an AVANCE III FT-NMR spectrometer (Bruker BioSpin, Rheinstetten, Germany) equipped with a 5 mm BBFO probe. The chemical shifts were referenced to residual ^1^H or ^13^C signals of the solvents: acetone-*d*_6_ (δ_H_ 2.05; δ_C_ 29.84) or methanol-*d*_4_ (δ_H_ 3.31; δ_C_ 49.00). The UV spectra were acquired using a V-550 spectrometer (Jasco, Tokyo, Japan); the samples for UV spectroscopy were dissolved in MeOH. LC-MS and LC-MS/MS were performed with a 3200 QTRAP LC/MS/MS system (SCIEX, Framingham, MA, USA) coupled with a Prominence UFLC system (Shimadzu Co., Kyoto, Japan). The FAB-MS were recorded with a JMS-BU25 mass spectrometer (Jeol, Tokyo, Japan) in the negative ion mode; glycerol was used as the matrix and argon was used as the FAB gas. Polyethylene glycol was used as an internal standard for HRMS analysis. GC-MS was performed with a JMS-BU25 mass spectrometer coupled with an HP6890 gas chromatograph (Agilent Technologies, Santa Clara, CA, USA). Preparative HPLC was carried out with PU-980 HPLC pumps and an MD-910 photodiode array detector (Jasco).

### 3.2. Chemicals

Sakuranetin (**1**) was chemically synthesized from naringenin (**2**; Sigma-Aldrich, St. Louis, MO, USA) according to a previously reported method [16]. The sakuranetin and naringenin used in this study were racemic mixtures. d-Xylose was purchased from Tokyo Chemical Industry (Tokyo, Japan). d-Ribose was purchased from Wako Pure Chemical Industries (Osaka, Japan). l-Arabinose was purchased from Nacalai Tesque (Kyoto, Japan). A Sylon BFT kit (BSTFA + TMCS, 99:1; Supelco, Bellefonte, PA, USA) was used for trimethylsilylation.

### 3.3. Fungal Material

The rice sheath blight fungus (*Rhizoctonia solani* MAFF305003) was obtained from NARO Genebank Project (Tsukuba, Japan) and was maintained on a potato dextrose agar (PDA) medium (Nissui Pharmaceutical, Tokyo, Japan) as a stock culture. A small portion of this stock culture was inoculated and grown on PDA in a Petri dish (9 mm in diameter) at 26 °C in the dark prior to use in the following experiments.

### 3.4. Incubation of the Rhizoctonia solani Suspension Cultures with Sakuranetin *(**1**)*

A portion of approximately 1 × 1 cm of the fungal layer was excised from the 5 dpi PDA medium. The fungal layer was homogenized using a spatula and suspended in 50 mL of potato dextrose broth (PDB; Sigma-Aldrich). The fungal culture was incubated for 3 days in the dark at 27 °C with rotary shaking at 150 rpm. A spherical mycelial cluster (approximately 5 mm in diameter) that was formed was transferred to fresh PDB (1 mL) to which 7.0 mmol/L **1** in MeOH (15 µL) had been added. The medium was incubated for 3–24 h at 27 °C with reciprocal shaking at 200 strokes/min.

### 3.5. Screening of the Sakuranetin *(**1**)* Metabolites from the Rhizoctonia solani Suspension Culture

Following the addition of **1**, the medium (1 mL) was incubated for 0 or 12 h and then diluted with MeOH (8 mL). The extract was filtered through a cotton-plugged Pasteur pipette. The filtrate was evaporated to dryness in vacuo, the residue was dissolved in 800 µL of MeOH, and the resulting solution was filtered through a 0.22-µm membrane filter. A 10-µL aliquot of the solution was subjected to LC-MS analysis to detect the putative metabolites of **1**. The Turbo V ion source was operated in the positive electrospray ionization (ESI) mode. LC separation of the analytes was achieved on a Unison UK-18 column (150 × 2.0 mm i.d., 3.0 µm particle size; Imtact Co., Kyoto, Japan) with a binary gradient of 0.1% (*v*/*v*) aqueous HCOOH (solvent A) and MeOH containing 0.1% HCOOH (solvent B) at a flow rate of 0.2 mL/min at 40 °C. The solvent gradient elution was performed with the following program: (i) initial, 20% B; (ii) 0–5 min, isocratic elution with 20% B; (iii) 5–25 min, a linear gradient from 20% B to 100% B; and (iv) 25–30 min, isocratic elution with 100% B. The following parameters were used for the ion source and MS: (i) curtain gas (CUR), 20 psi; (ii) temperature (TEM), 450 °C; (iii) nebulizer gas (GS1), 50 psi; (iv) GS2, 50 psi; (v) ion spray voltage (IS), 5200 V; (vi) declustering potential (DP), 51.0 V; and (vii) entrance potential (EP), 7.5 V. The scan range for the MS anaysis was set to *m*/*z* 100–700 (enhanced MS scan).

### 3.6. LC-MS/MS Analysis to Identify Naringenin *(**2**)* as a Metabolite

LC-ESI-CID-MS analysis was performed according to a previously described method [10].

### 3.7. Purification of Sakuranetin-4′-O-β-d-xylopyranoside *(**3**)* and Naringenin-7-O-β-d-xylopyranoside *(**4**)* from the Rhizoctonia solani Suspension Culture

A portion of approximately 1 × 1 cm of the fungal layer was excised from the 5 dpi PDA medium. The fungal layer was homogenized using a spatula and suspended in PDB (300 mL). The fungal culture was incubated for 5 days in the dark at 27 °C with rotary shaking at 150 rpm. After the addition of 35 mmol/L **1** in MeOH (1 mL), the culture was incubated for 18 h under the same conditions. MeOH (200 mL) was added to the culture, and the mixture was homogenized with a Physcotron homogenizer (Microtec, Funabashi, Japan). The homogenate was filtered through filter paper, and the filtrate was concentrated in vacuo. The concentrate (250 mL) was extracted with EtOAc (250 mL × 3) and the organic layer was concentrated to dryness in vacuo. The residue of the EtOAc extract was dissolved in MeOH to a concentration of 4% (*v*/*v*) and separated on a Luna C18(2) column (25 × 1 cm i.d., 5 µm particle size, Phenomenex, Torrance, CA, USA) with a binary gradient of H_2_O (solvent A) and MeOH (solvent B) at a flow rate of 2 mL/min at 40 °C. The gradient elution was performed with the following program: (i) initial, 40% B; (ii) 0–50 min, isocratic elution with 40% B; (iii) 50–80 min, a linear gradient from 40% B to 70% B; and (iv) 80–100 min, isocratic elution with 40% B. The injection volume in each run was 50 µL, and the sample was separated repeatedly under the same conditions. The detection wavelength was 280 nm. The presence of **3** and **4** was confirmed using LC/MS under the same conditions as were used in the screening of the metabolites. The peak containing **3** (*t*_R_ 77–78 min) was collected and evaporated to dryness in vacuo to afford 1.1 mg of **3**. The peak containing **4** (*t*_R_ 43–46 min) was collected and evaporated to dryness in vacuo to afford 2.4 mg of **4**.

*Sakuranetin-4′-O-β-d-xylopyranoside* (**3**). HRMS (FAB): *m*/*z* 417.1172 ([M − H]^−^); calcd. for C_21_H_21_O_9_, 417.1185. ^1^H and ^13^C NMR (methanol-*d*_4_): see Table 1; UV (MeOH) λ_max_ (log ε): 288 (4.0).

*Naringenin-7-O-β-d-xylopyranoside* (**4**). HRMS (FAB): *m*/*z* 403.1042 ([M − H]^−^); calcd. for C_20_H_19_O_9_, 403.1029. ^1^H and ^13^C NMR (acetone-*d*_6_): see Table 1. UV (MeOH) λ_max_ (log ε): 283 (4.3).

### 3.8. GC-MS Analysis of the Hydrolysates of Sakuranetin-4′-O-β-d-xylopyranoside *(**3**)* and Naringenin-7-O-β-d-xylopyranoside *(**4**)*

The hydrolysis reaction was performed according to a previous study [17]. Compound **3** or **4** (1 µg) was hydrolyzed with 40 µL of 2 mol/L TFA at 100 °C for 2 h. The hydrolysate mixture was concentrated to dryness in vacuo. The residue was trimethylsilylated with 50 µL of BSTFA + TMCS (99:1) at 70 °C for 3 h. The trimethylsilylated sample (2 µL) was subjected to GC-MS analysis. GC separation was carried out on a Zebron ZB-5MS column (30 m × 0.25 mm i.d., 0.25 µm film thickness, Phenomenex) under the following conditions: injector temperature, 280 °C; carrier gas, helium; and flow rate, 1.0 mL/min. The temperature program of the column oven was set to hold at 70 °C for 1 min, then increase at 10 °C/min to 300 °C, and finally hold at 300 °C for 3 min. The conditions used for the mass spectrometer were as follows: ionization mode, EI (70 eV); ion source temperature, 200 °C; scan range, *m*/*z* 61–760; and scan rate, 1 s/scan.

### 3.9. Quantitation of Sakuranetin *(**1**)* and Its Metabolites in the Rhizoctonia solani Suspension Culture

After incubation, MeOH (8 mL) was added to the medium (1 mL), and the resulting suspension was shaken for 1 h. The extract was filtered through a cotton-plugged Pasteur pipette and a membrane filter (0.22 µm). A portion of the filtrate (0.1 mL) was diluted with MeOH (0.9 mL), and 2 µL of the dilution was subjected to LC-MS/MS analysis. The Turbo V ion source was operated in the negative ESI mode. LC separation of the analytes was achieved on a TSK-gel ODS-100V column (50 × 2.0 mm i.d., 3.0 µm particle size; Tosoh Corp., Tokyo, Japan) with a binary gradient of 0.1% (*v*/*v*) aqueous HCOOH (solvent A) and MeOH containing 0.1% HCOOH (solvent B) at a flow rate of 0.2 mL/min at 40 °C. The solvent gradient elution was performed according to the following program: (i) initial, 20% B; (ii) 0–1 min, isocratic elution with 20% B; (iii) 1–6 min, a linear gradient from 20% B to 100% B; and (iv) 6–9 min, isocratic elution with 100% B. The following parameters were used for the ion source: (i) CUR, 10 psi; (ii) TEM, 300 °C; (iii) GS1, 30 psi; (iv) GS2, 80 psi; and (v) IS, −4500 V. The selective reaction monitoring (SRM) transitions and MS parameters were optimized to detect **1**–**4** using Analyst 1.6.2 software (SCIEX). The following SRM transitions (*t*_R_) were monitored: (**1**) *m*/*z* 285 → 119 (7.3 min), (**2**) *m*/*z* 271 → 151 (6.6 min), (**3**) *m*/*z* 417 → 285 (6.9 min), and (**4**) *m*/*z* 403 → 271 (6.1 min). The optimized MS parameters for each compound were as follows: (**1**) −45 V DP, −4.5 V EP, and −24 V CE; (**2**) −45 V DP, −6.5 V EP, and −22 V CE; (**3**) −60 V DP, −3 V EP, and –18 V CE; and (**4**) −40 V DP, −3.5 EP, and −22 CE. Calibration curves were prepared using the SRM peak areas of standards for **1** and **2**–**4** in concentration ranges of 20–2000 ng/mL and 10–1000 ng/mL, respectively.

### 3.10. Assay of the Antifungal Activity Against Rhizoctonia solani

The Fungal colony growth inhibition assay was performed according to a previously described method, which used *Pyricularia oryzae* as the test fungus [18]. In this study, *R. solani* was used instead of *P*. *oryzae*.

## 4. Conclusions

Xylosylated flavanones sakuranetin-4′-*O*-β-d-xylopyranoside (**3**) and naringenin-7-*O*-β-d-xylopyranoside (**4**) as well as naringenin (**2**) were identified as detoxified metabolites of sakuranetin (**1**) by the rice sheath blight fungus *Rhizoctonia solani*. Compound **2** is a common detoxified metabolite of **1** by both *R*. *solani* and *Pyricularia oryzae*. However, xylosylation by *R*. *solani* is a rare and efficient detoxification path of phytoalexins.

## Figures and Tables

**Table 1:** ^13^C and ^1^H NMR data for compounds **3** (methanol-*d*_4_) and **4** (acetone-*d*_6_).

Position	Compound 3		Compound 4	
	δ_C_	δ_H_ (Multiplicity, *J* in Hz)	δ_C_	δ_H_ (Multiplicity, *J* in Hz)
2	80.2	5.45 (dd, 12.7, 3.1)	80.15/80.17 ^1^	5.50 (dd, 13.0, 3.2)
3	44.1	2.79 (dd, 17.2, 3.1)	43.58/43.60 ^1^	2.78 (m) ^2^
		3.14 (dd, 17.2, 12.7)		3.24/3.25 ^1^ (dd, 17.0, 13.0)
4	197.9		197.97/197.99 ^1^	
5	165.3		164.72/164.76 ^1^	
6	95.8	6.05 (d, 2.3)	97.6	6.11/6.12 ^1^ (d, 2.2)
7	169.6		166.46/166.53 ^1^	
8	95.0	6.08 (d, 2.3)	96.4	6.15 (d, 2.2)
9	164.6		164.2	
10	104.1		104.5	
7-*O*-Me	56.3	3.81 (s)	–	–
5-OH	–	–		12.07
1′	134.2		130.63/130.67 ^1^	
2′, 6′	128.8	7.44 (d, 8.7)	129.10/129.14 ^1^	7.41 (d, 8.7)
3′, 5′	117.9	7.11 (d, 8.7)	116.3	6.91 (d, 8.7)
4′	159.2		158.84/158.85 ^1^	
1″	102.8	4.90 (d, 7.2)	101.39/101.46 ^1^	5.06/5.07 ^1^ (d, 7.0 or 5.8) ^3^
2″	74.7	3.44 (m)^2^	74.1	3.48 (m) ^2^
3″	77.7	3.44 (m)^2^	77.4	3.48 (m) ^2^
4″	71.0	3.57 (m)	70.6	3.60 (m)
5″	66.9	3.37 (dd, 11.4, 10.2)	66.6	3.50 (m) ^2^
		3.92 (dd, 11.4, 5.3)		3.91 (dd, 11.2, 4.8)

^1^ Each diastereomer showed different chemical shifts. ^2^ The chemical shifts were estimated by an HSQC experiment. ^3^ Two possibilities are listed for the *J* values; the true values could not be confidently assigned due to the overlapping signals of the two diastereomers.

**Table 2:** Inhibitory activity of **1**, **2**, **3**, and **4** on *Rhizoctonia solani* mycelium growth after 48 h of incubation.

Compound	Concentration (µmol/L)		
	75	150	300
	Length of Colony [mm] (Inhibition [%])		
**1**	9.8 ± 0.6 (31)	8.1 ± 0.7 (42)	6.8 ± 1.2 (52)
**2**	14.5 ± 0.6 (−3)	11.3 ± 0.2 (20)	10.6 ± 0.5 (25)
**3**	12.8 ± 0.2 (10)	13.1 ± 0.9 (7)	12.9 ± 0.2 (9)
**4**	14.4 ± 0.2 (−2)	14.0 ± 0.2 (1)	14.0 ± 0.8 (1)

Values are presented as the mean ± SD (*n* = 4). Inhibition [%] = (1-colony length of sample/colony length of control) × 100. Colony length of the control was 14.1 ± 1.0 mm.

## Supplementary Materials

Supplementary materials are available online.
