# Supplementary material for: Xylosylated Detoxification of the Rice Flavonoid Phytoalexin Sakuranetin by the Rice Sheath Blight Fungus Rhizoctonia solani
Source: Molecules. 2018 Jan 29;23(2):276. doi: 10.3390/molecules23020276 (PMC6017487; doi:10.3390/molecules23020276)
Supplement: Supplementary file 1 [file molecules-23-00276-s001.pdf]

molecules

Supplementary Materials

Xylosylated Detoxification of the Rice Flavonoid Phytoalexin Sakuranetin by the Rice Sheath Blight Fungus *Rhizoctonia solani*

Shun Katsumata, Hiroaki Toshima and Morifumi Hasegawa

Figure S1. Total ion current chromatograms obtained from the *Rhizoctonia solani* suspension culture without sakuranetin (**1**) and of the medium containing **1** without the fungus using LC-MS.

Figure S2. ESI-MS of peak I (t_R 17.4 min) and peak III (t_R 20.5 min) from the LC-MS analysis of the *Rhizoctonia solani* suspension culture containing sakuranetin (**1**).

Figure S3. GC-MS analysis of the TMS derivatives of the hydrolysate of **3**, **4**, and authentic D-xylose.

Figure S4. ^{1}H NMR data of **3**.

Figure S5. ^{13}C NMR data of **3**.

Figure S6. DEPT90 data of **3**.

Figure S7. DEPT135 data of **3**.

Figure S8. HSQC data of **3**.

Figure S9. COSY data of **3**.

Figure S10. TOCSY data of **3**.

Figure S11. HMBC data of **3**.

Figure S12. ^{1}H NMR data of **4**.

Figure S13. ^{13}C NMR data of **4**.

Figure S14. DEPT90 data of **4**.

Figure S15. DEPT135 data of **4**.

Figure S16. HSQC data of **4**.

Figure S17. COSY data of **4**.

Figure S18. TOCSY data of **4**.

Figure S19. HMBC data of **4**.

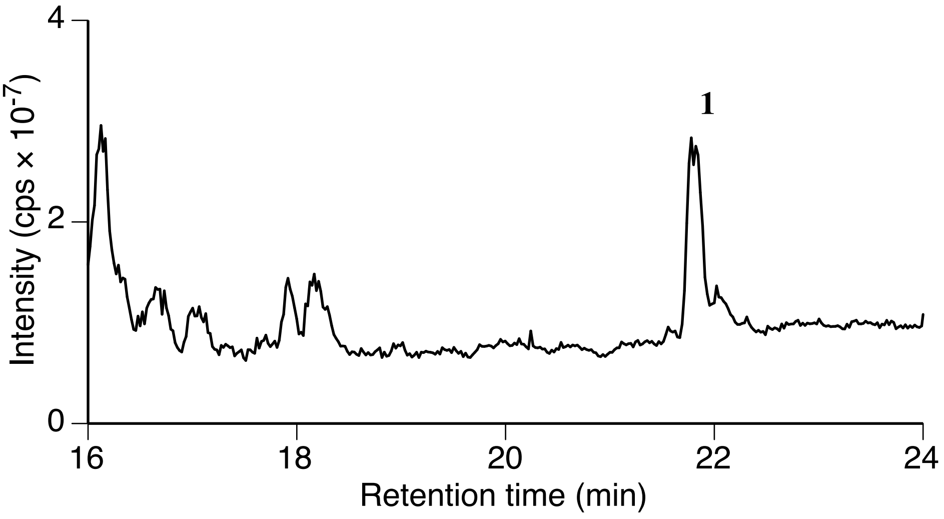

(**a**)

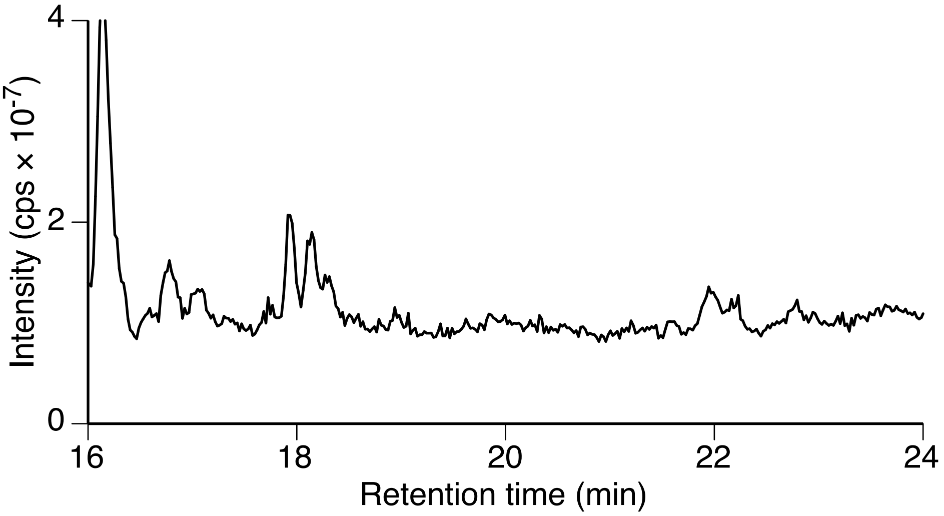

(**b**)

Figure S1. Total ion current chromatograms obtained from the *Rhizoctonia solani* suspension culture without sakuranetin (**1**) and of the medium containing **1** without the fungus using LC-MS. (**a**) The 12-h-incubated sample without *R. solani* containing **1**; (**b**) the 12-h-incubated sample with *R. solani* without **1**.

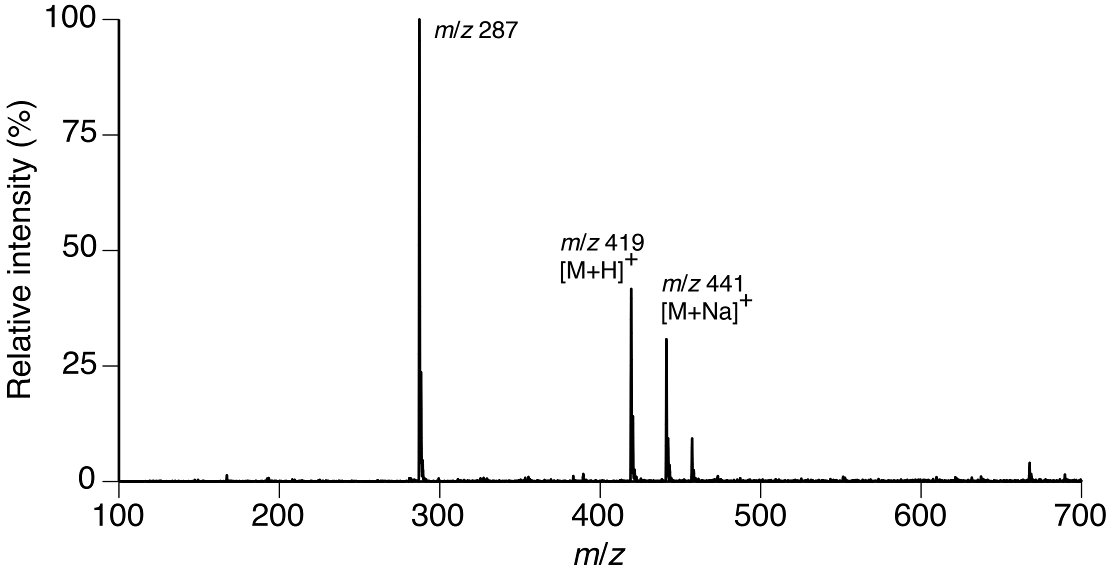

(**a**)

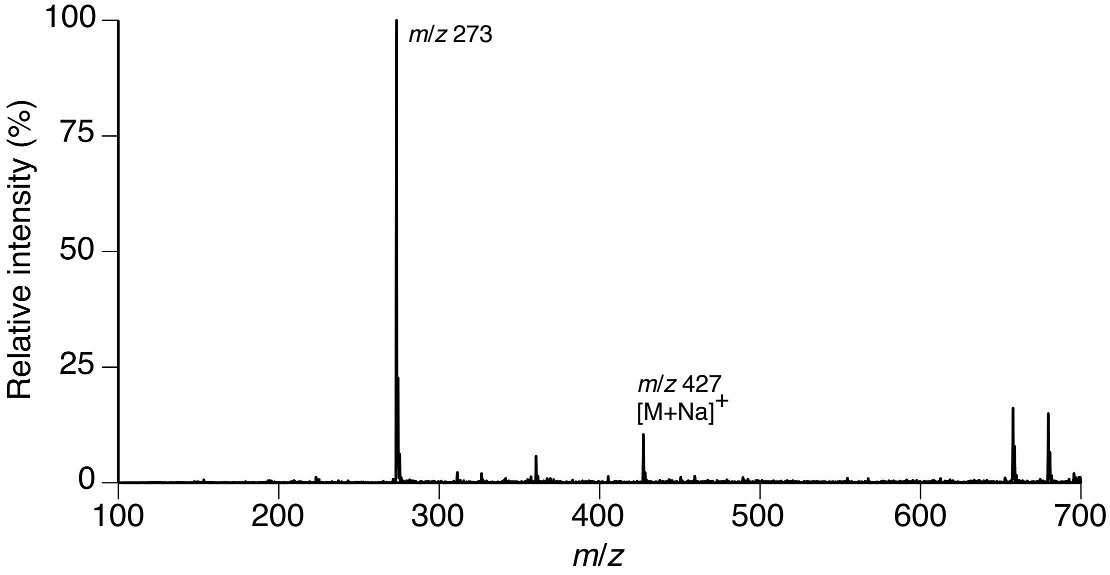

(**b**)

Figure S2. ESI-MS of peak I (t_R 17.4 min) and peak III (t_R 20.5 min) from the LC-MS analysis of the *Rhizoctonia solani* suspension culture containing sakuranetin (**1**). (**a**) peak I; (**b**) peak III.

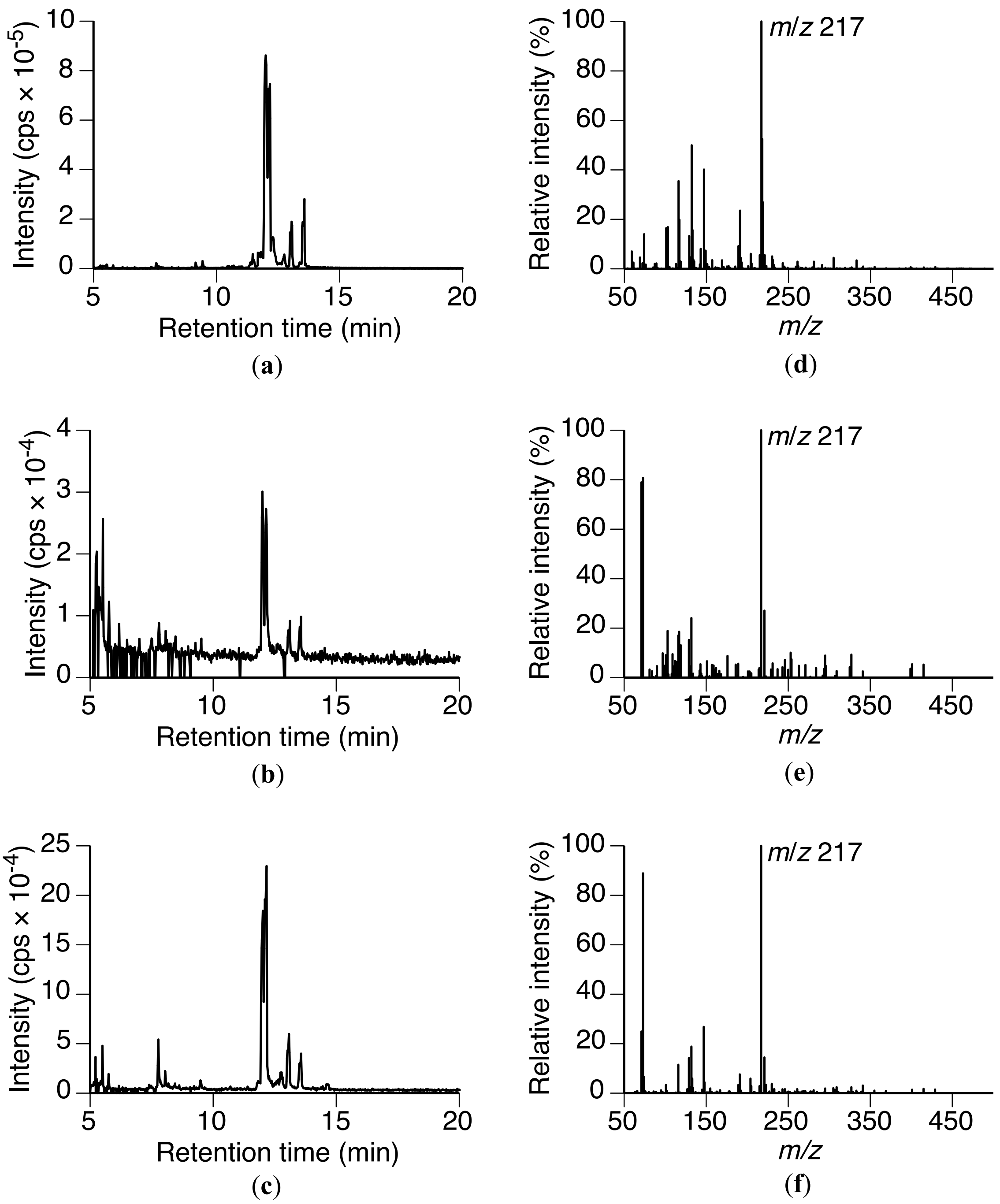

Figure S3. GC-MS analysis of the TMS derivatives of the hydrolysate of **3**, **4**, and authentic D-xylose. (**a**) Mass chromatogram at *m*/*z* 217 singal (base peak of TMS-D-xylose) of the TMS derivatives of authentic D-xylose; (**b**) mass chromatogram at *m*/*z* 217 signal of the TMS derivatives of the hydrolysate of **3**; (**c**) mass chromatogram at *m*/*z* 217 signal of the TMS derivatives of the hydrolysate of **4**; (**d**) mass spectrum of the peak at t_R 12.0 min of the TMS derivatives of authentic D-xylose; (**e**) mass spectrum of the peak at t_R 12.0 min of the TMS derivatives of the hydrolysate of **3**; (**f**) mass spectrum of the peak at t_R 12.0 min of the TMS derivatives of the hydrolysate of **4**.

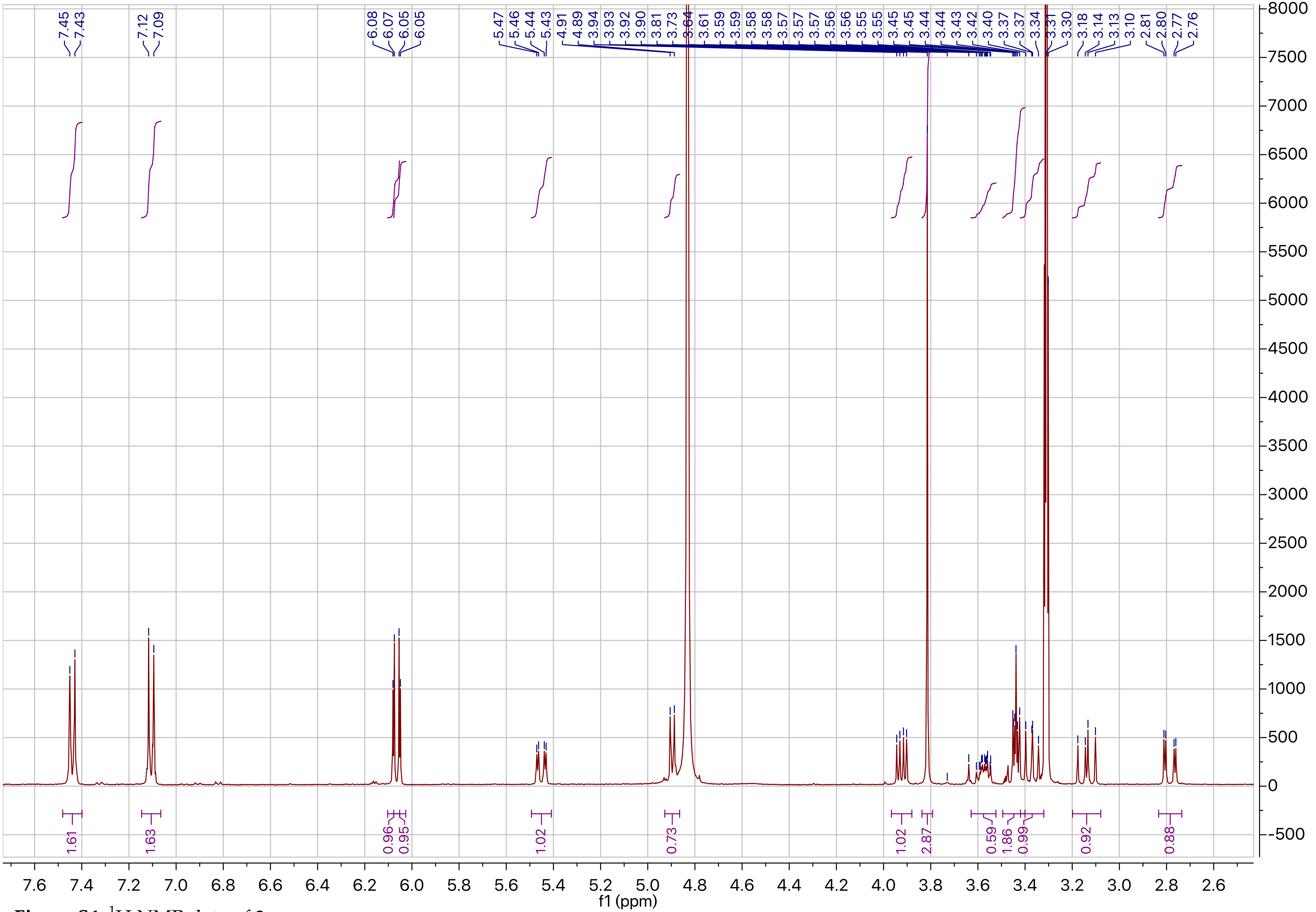

Figure S4. ^{1}H NMR data of **3**.

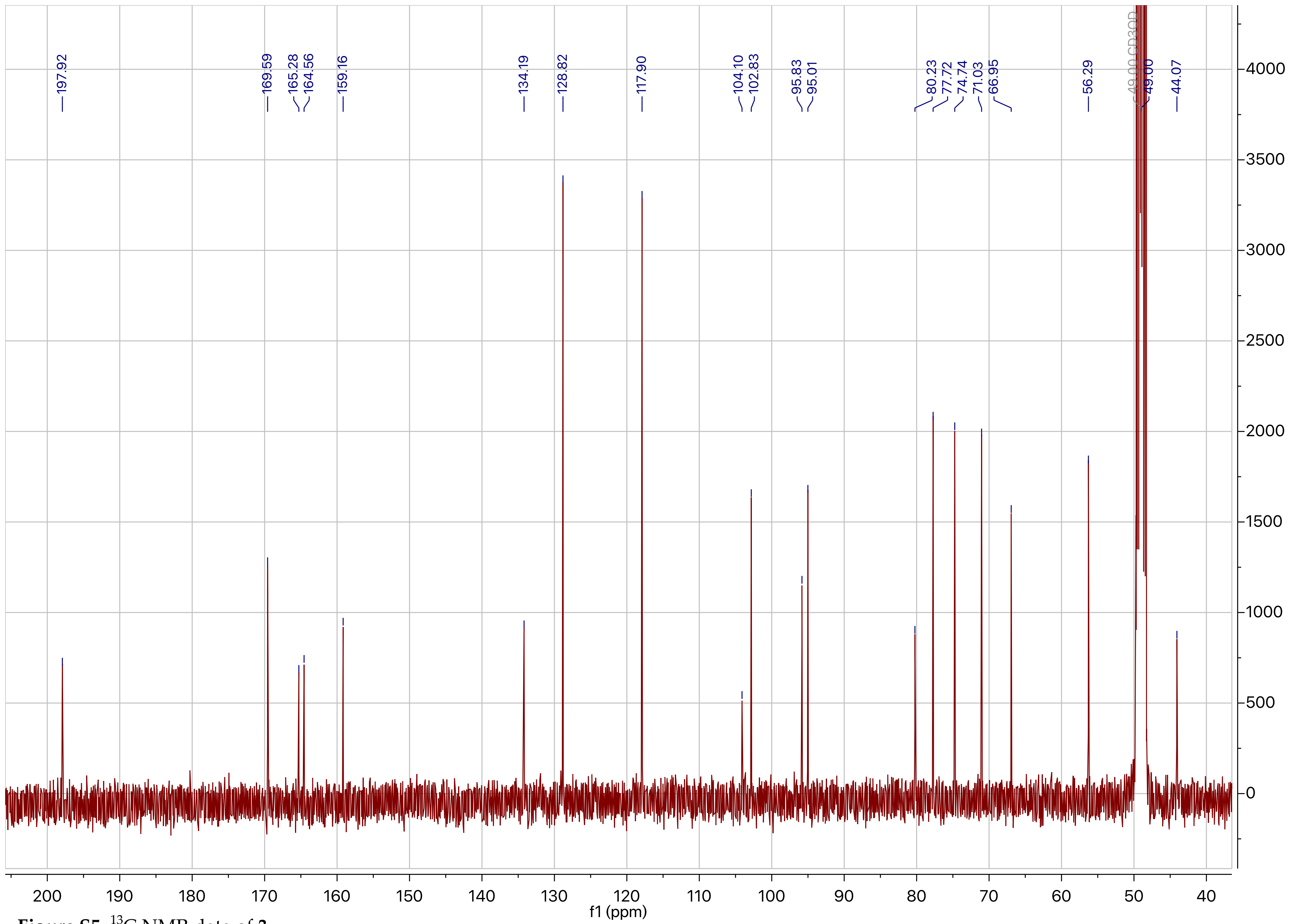

Figure S5. ^{13}C NMR data of **3**.

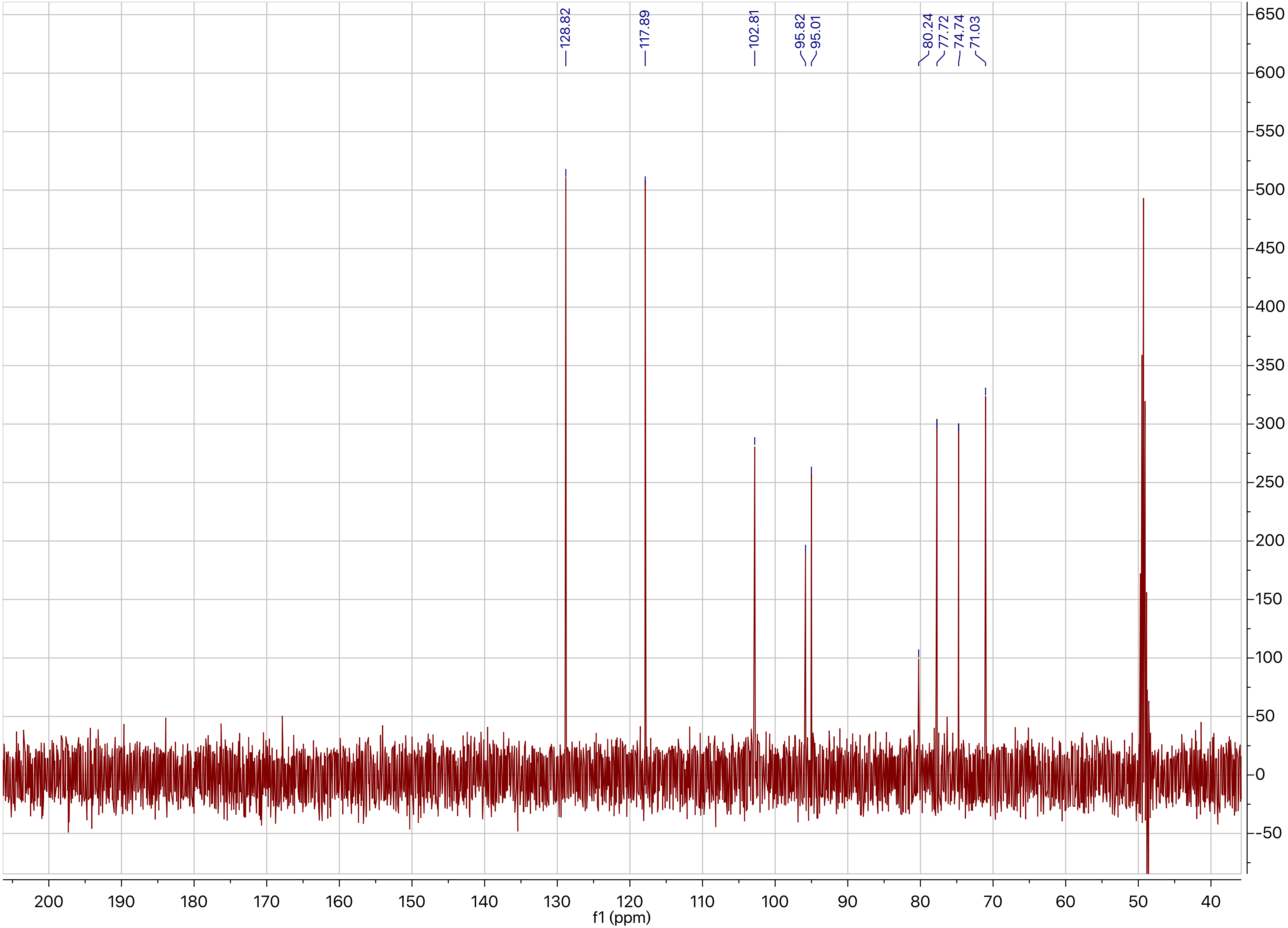

Figure S6. DEPT90 data of **3**.

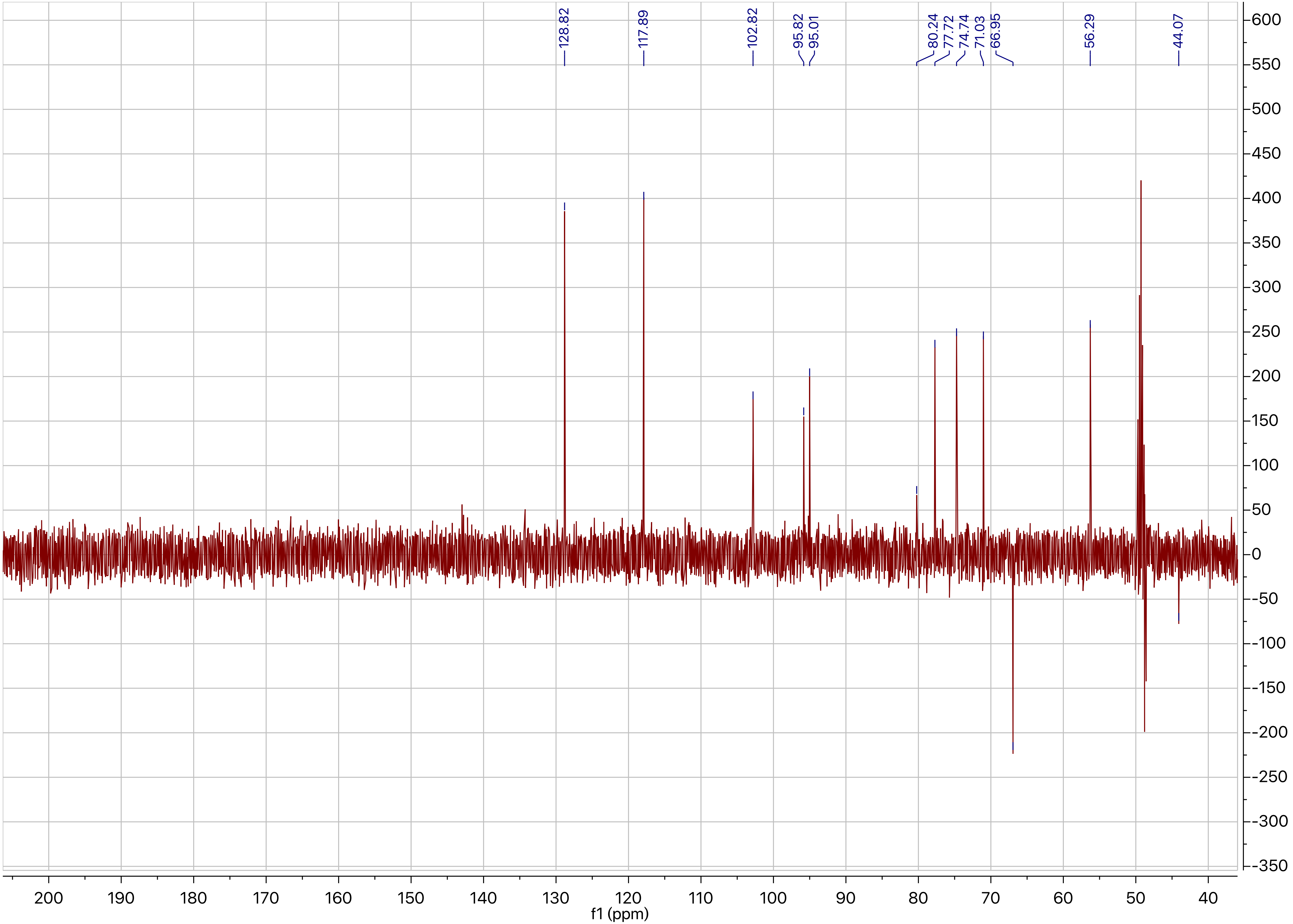

Figure S7. DEPT135 data of **3**.

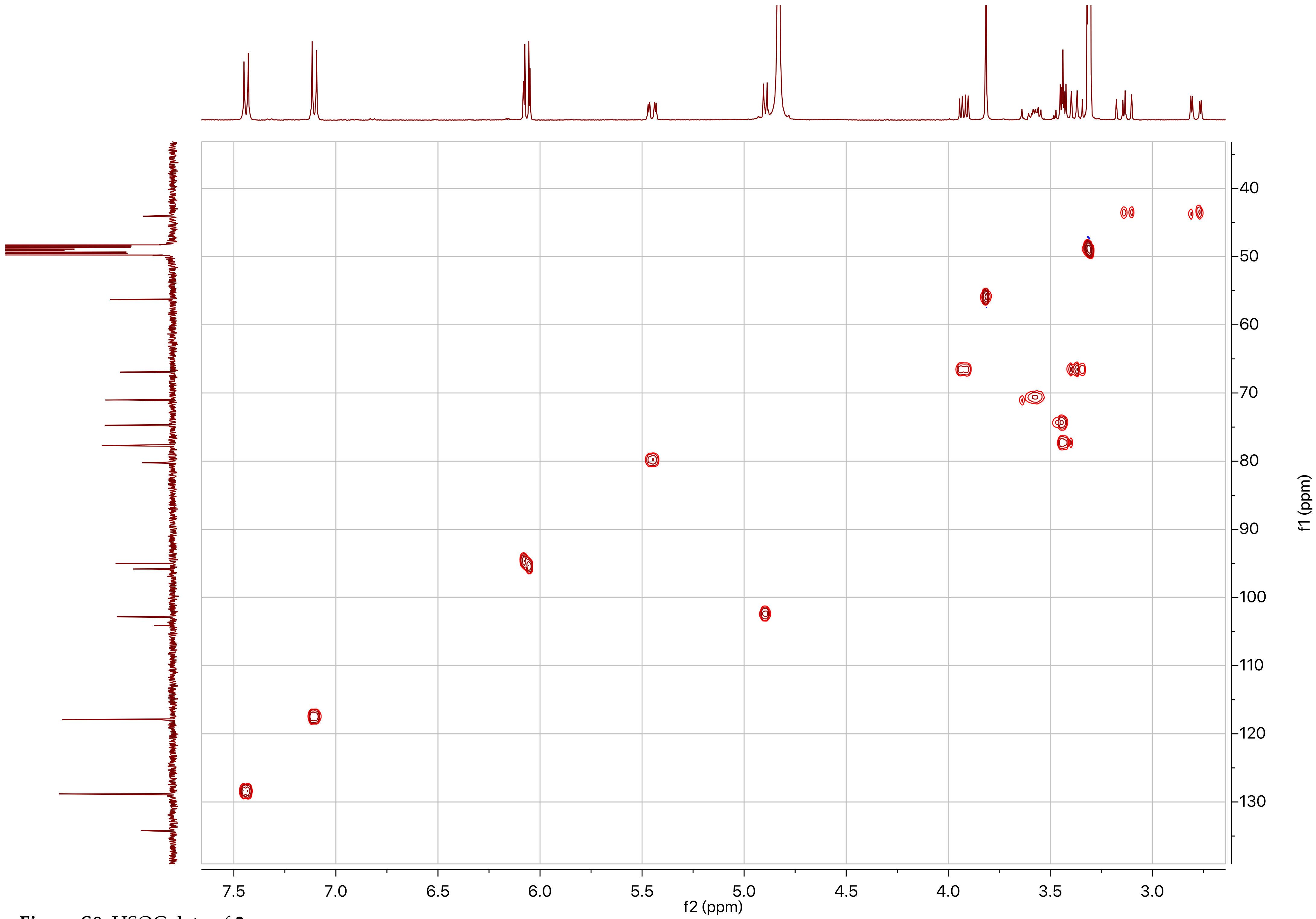

Figure S8. HSQC data of **3**.

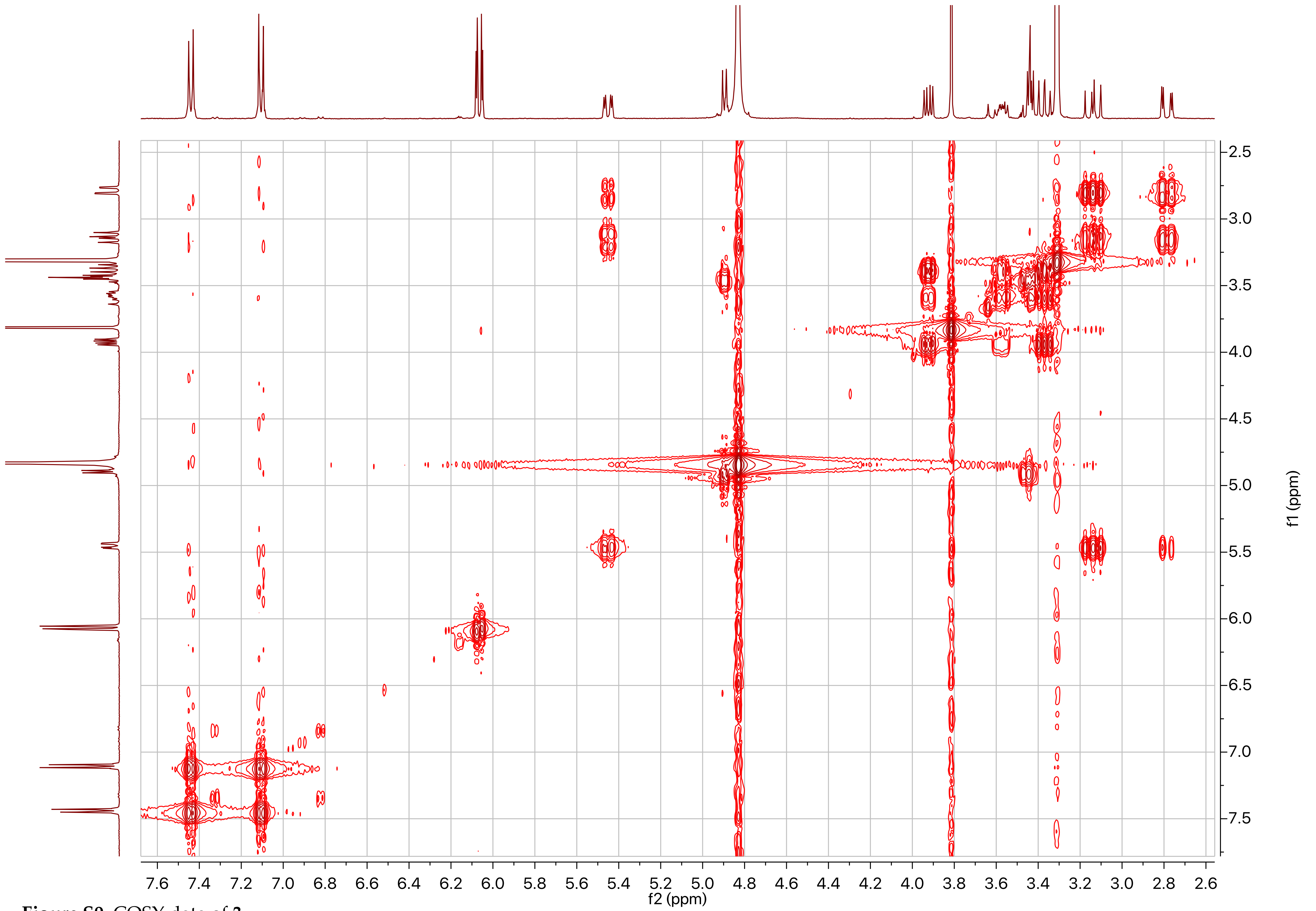

Figure S9. COSY data of **3**.

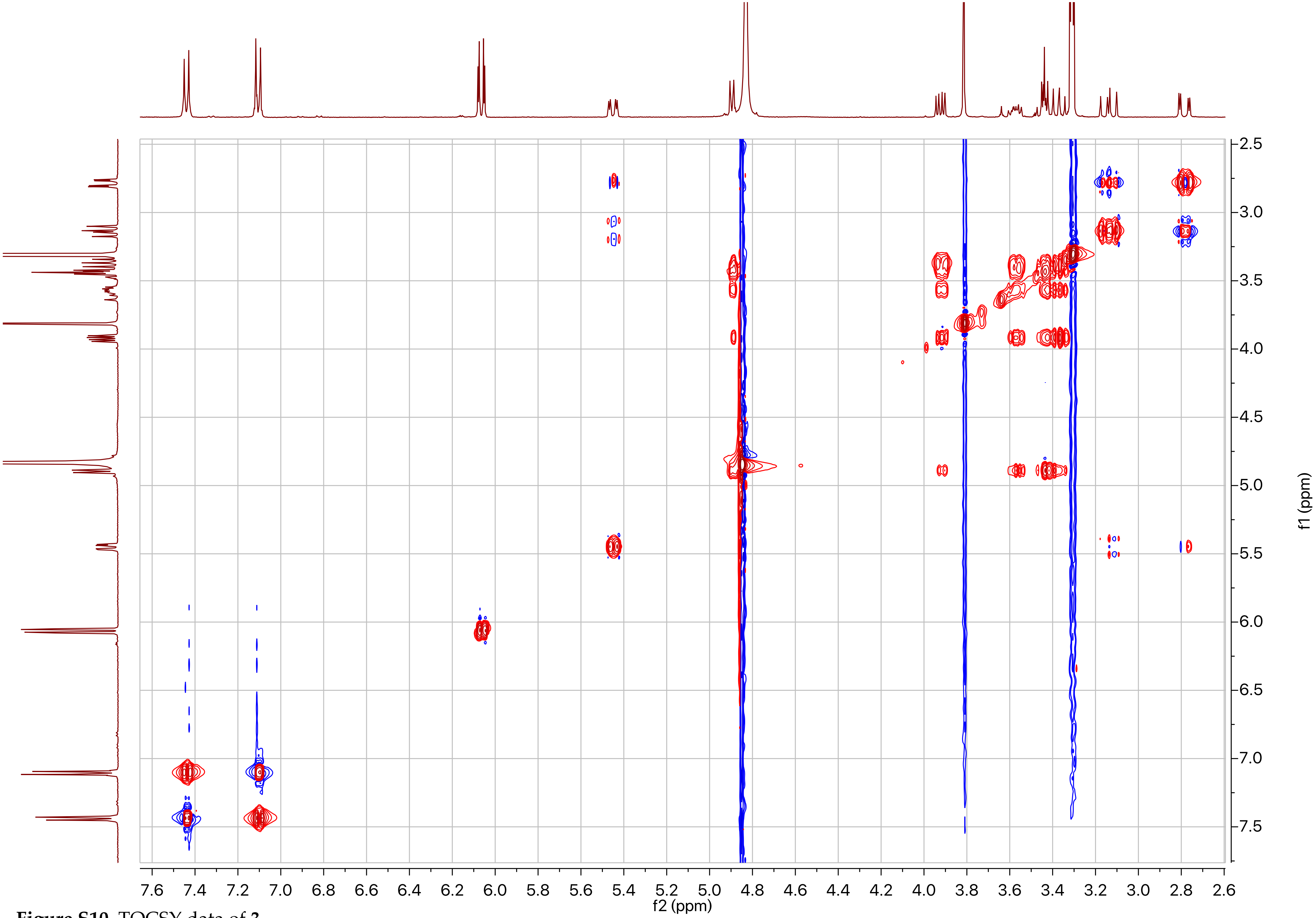

Figure S10. TOCSY data of **3**.

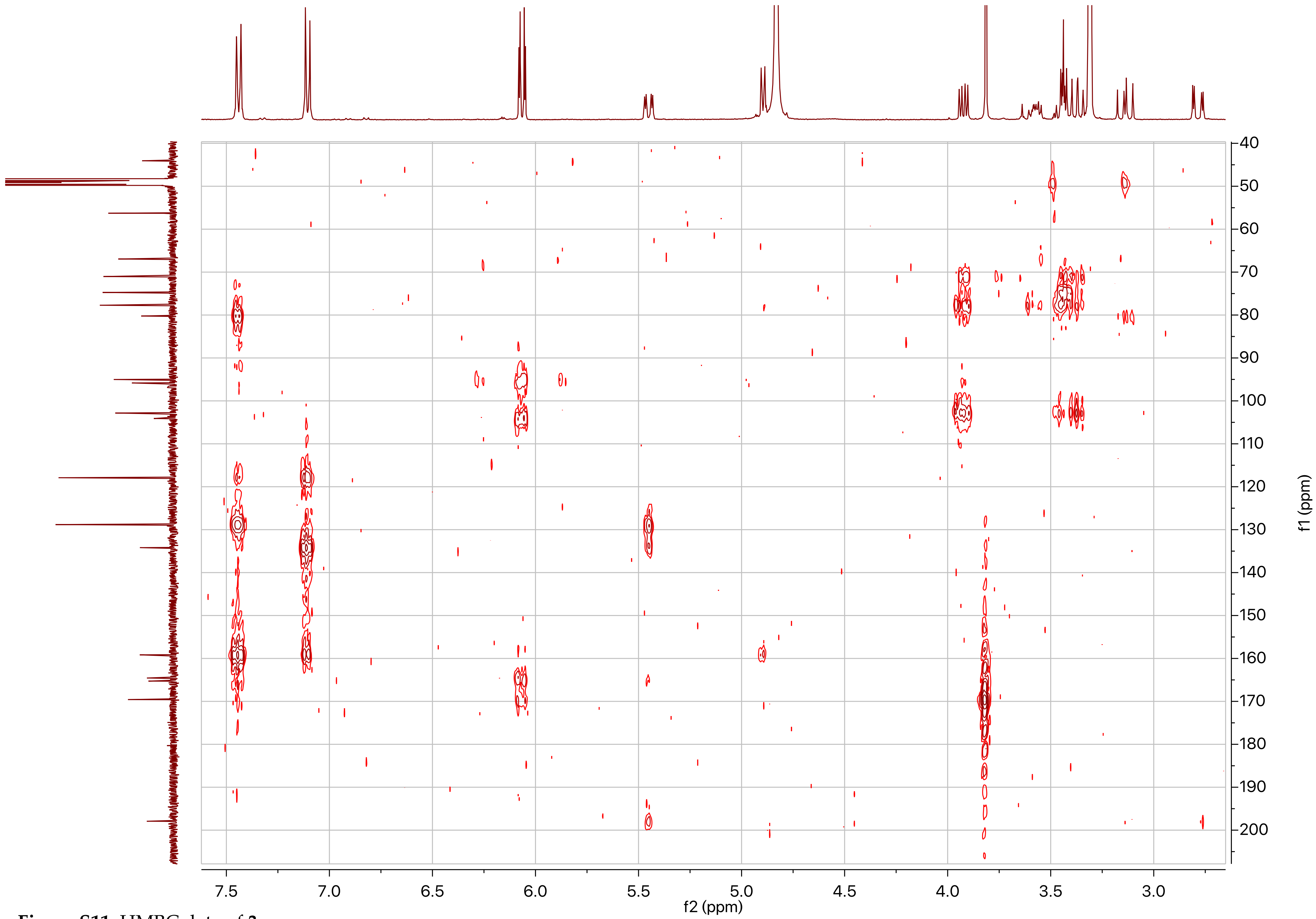

Figure S11. HMBC data of **3**.

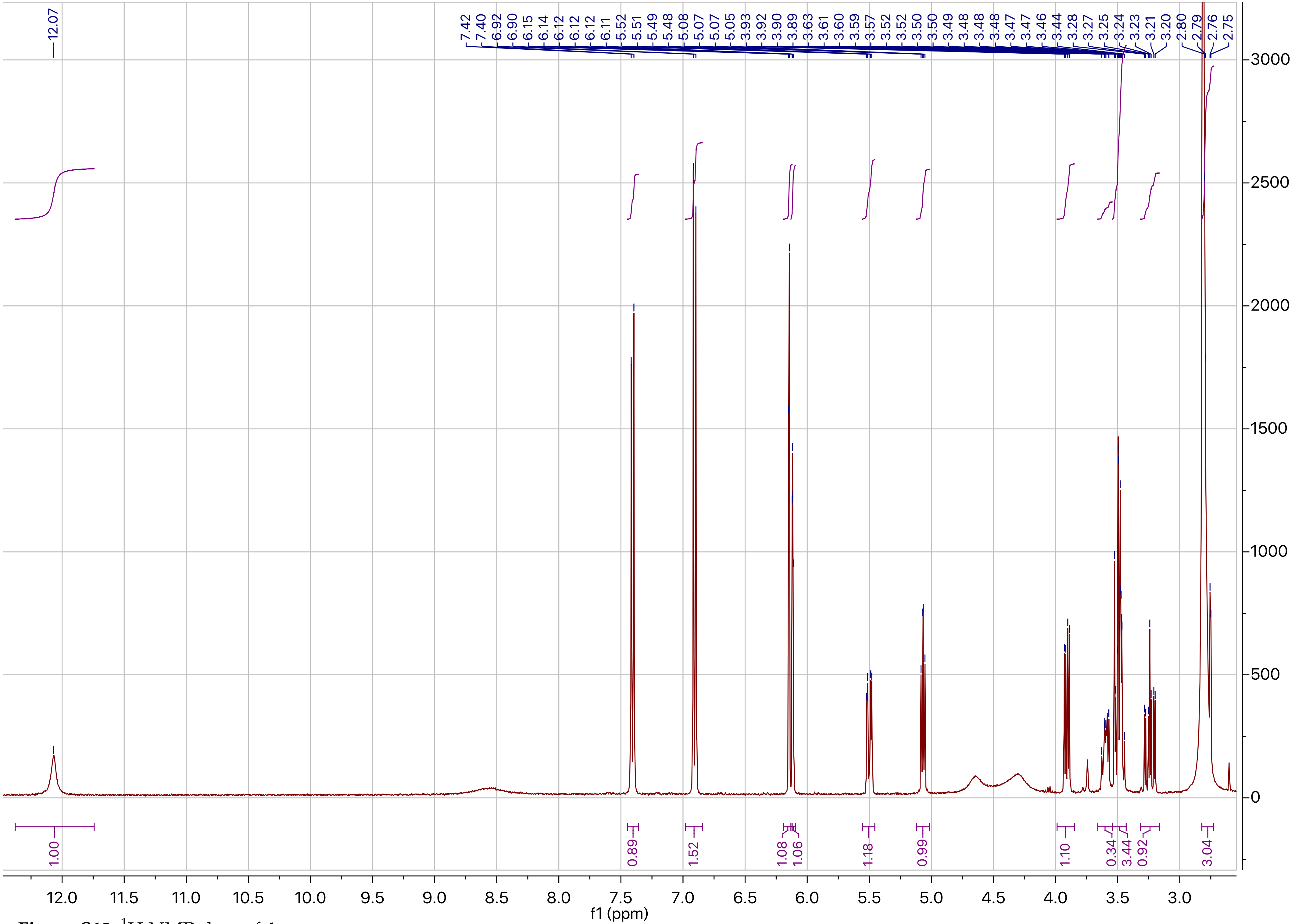

Figure S12. ^{1}H NMR data of **4**.

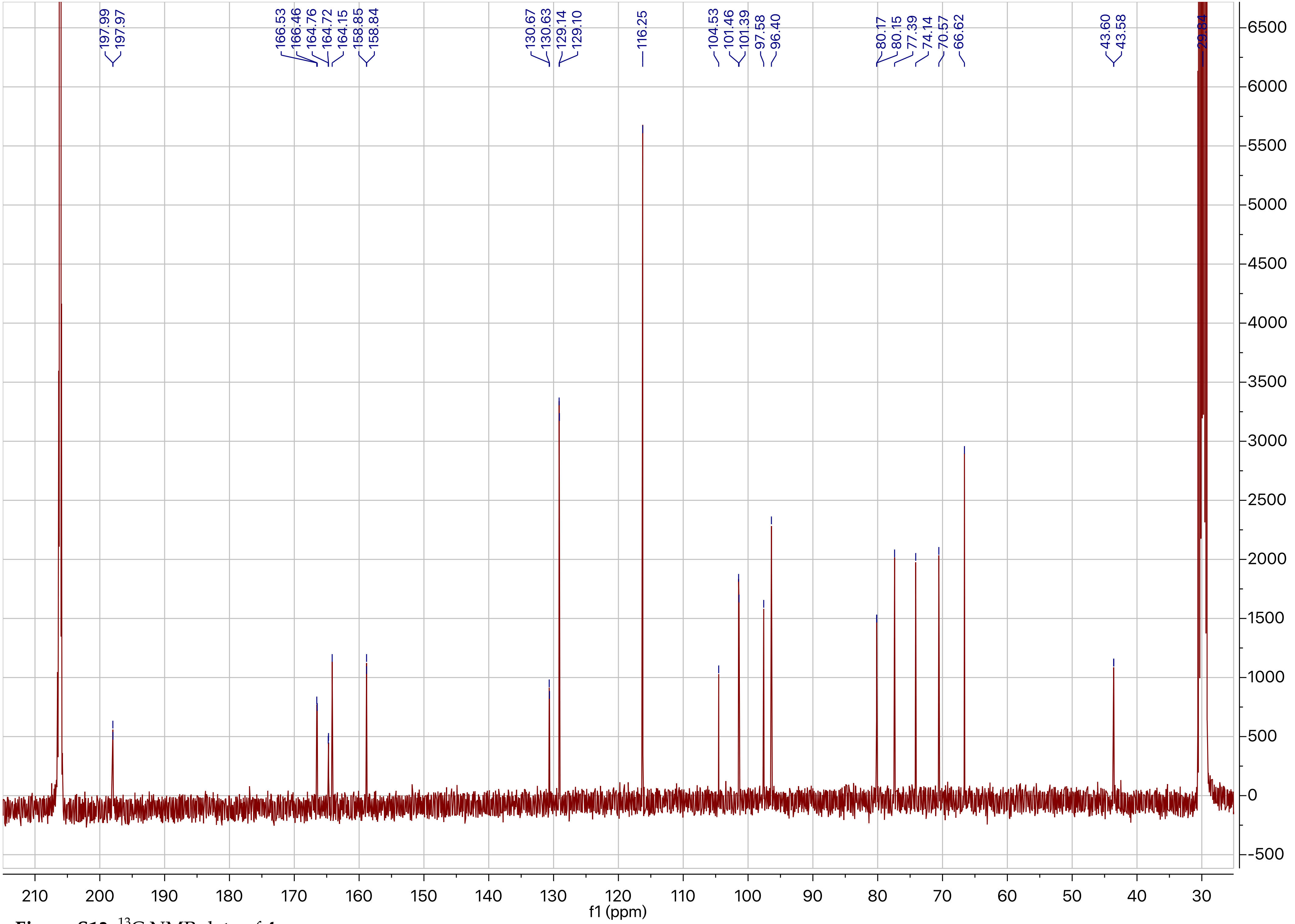

Figure S13. ^{13}C NMR data of **4**.

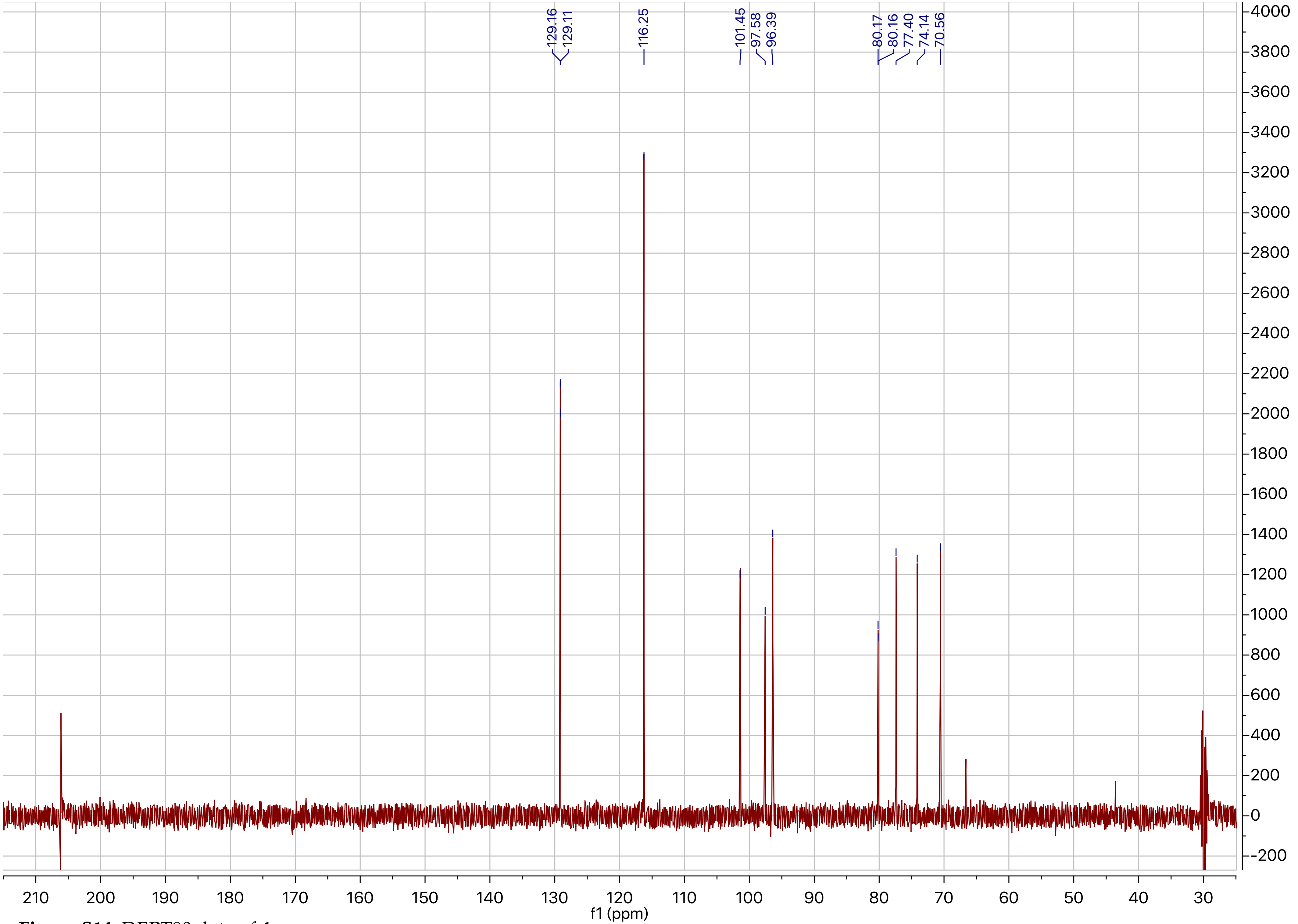

Figure S14. DEPT90 data of **4**.

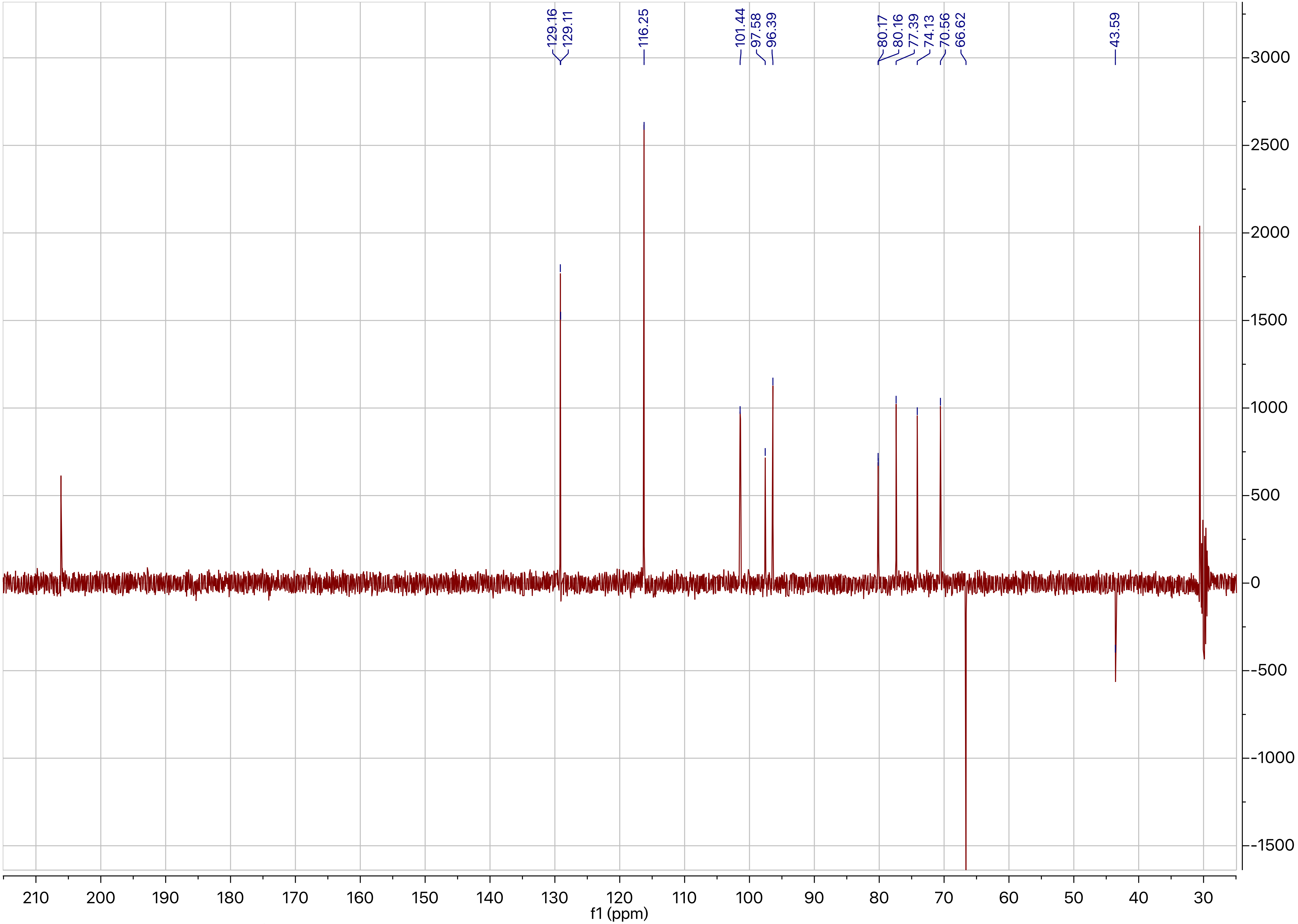

Figure S15. DEPT135 data of **4**.

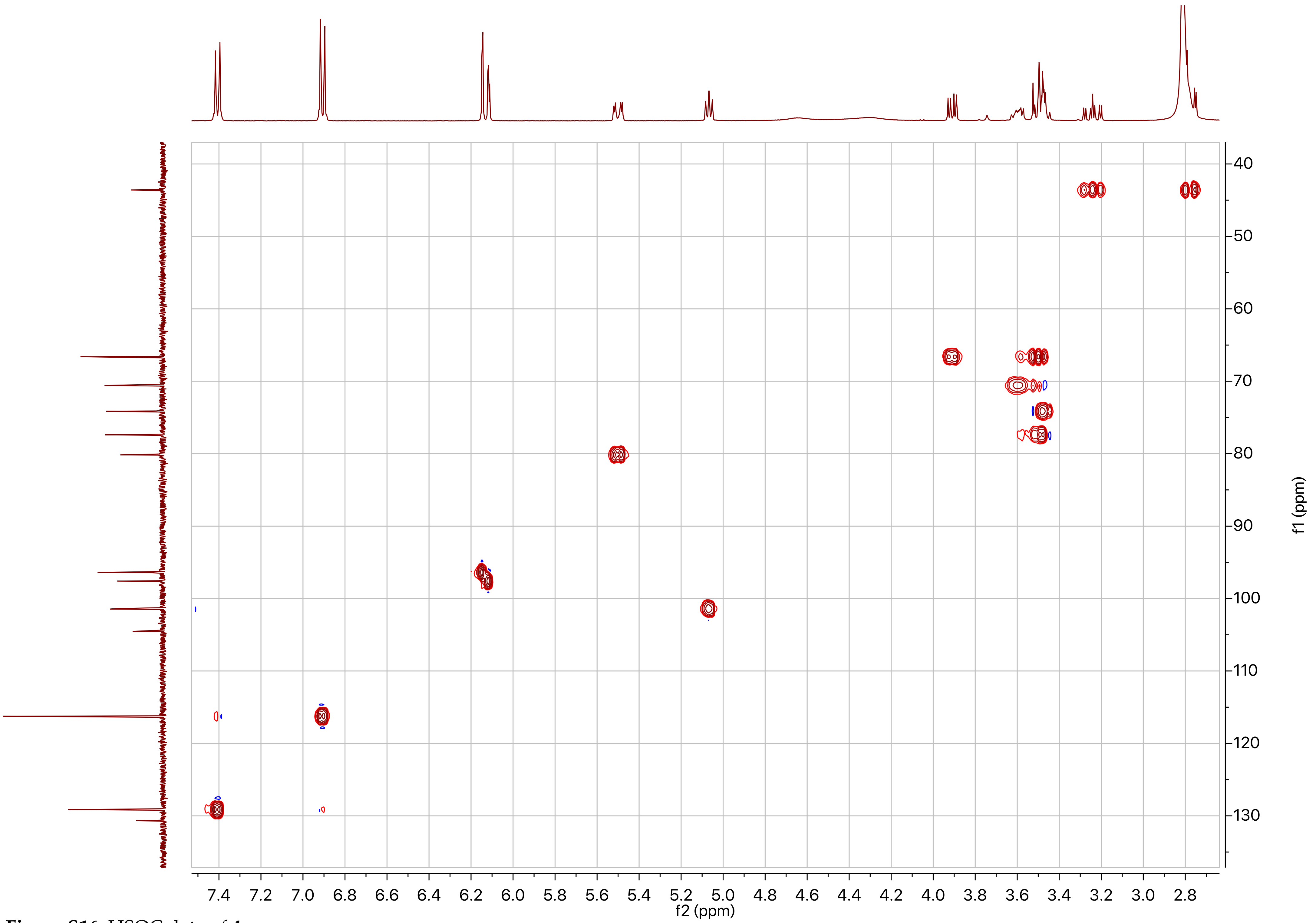

Figure S16. HSQC data of **4**.

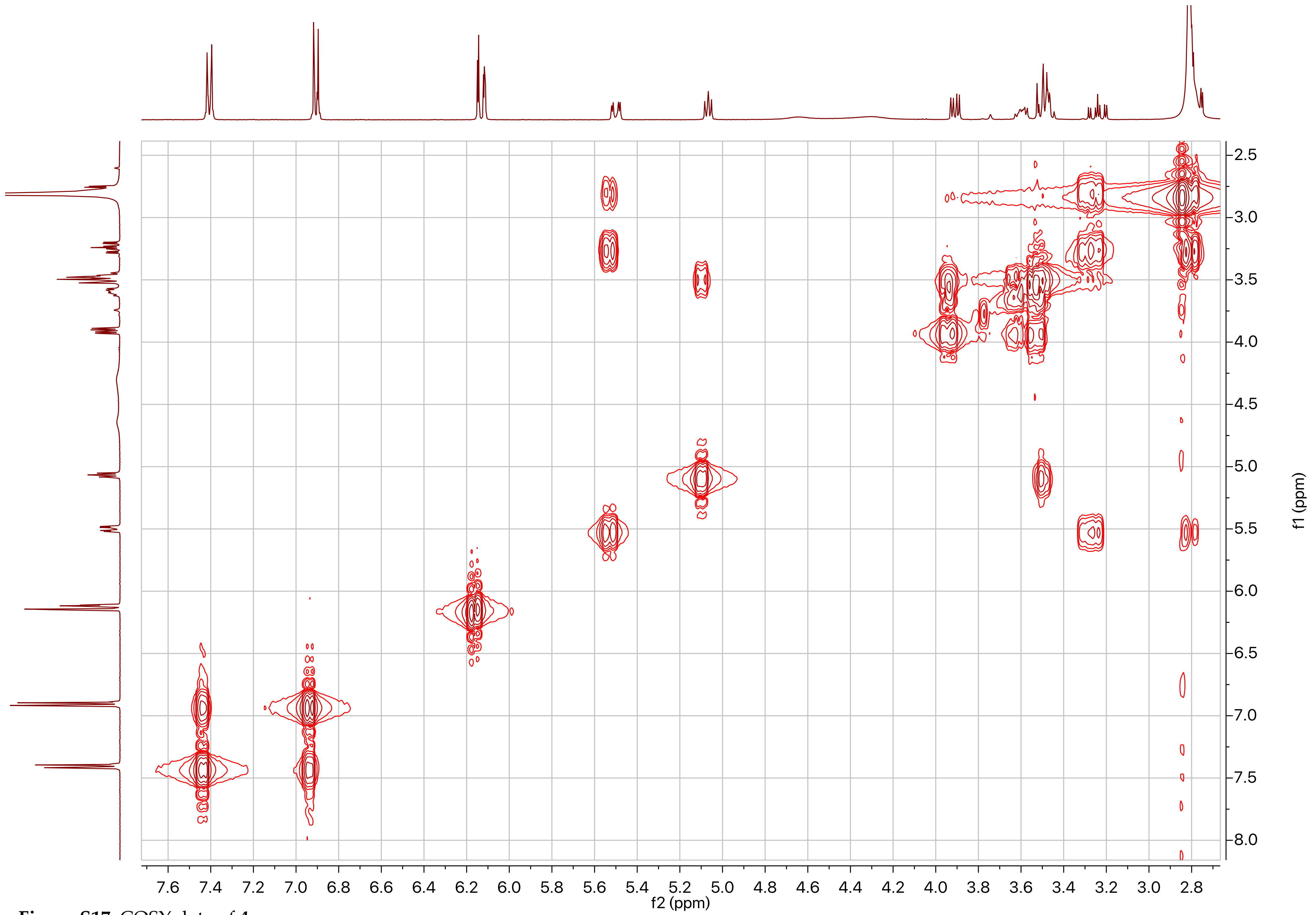

Figure S17. COSY data of **4**.

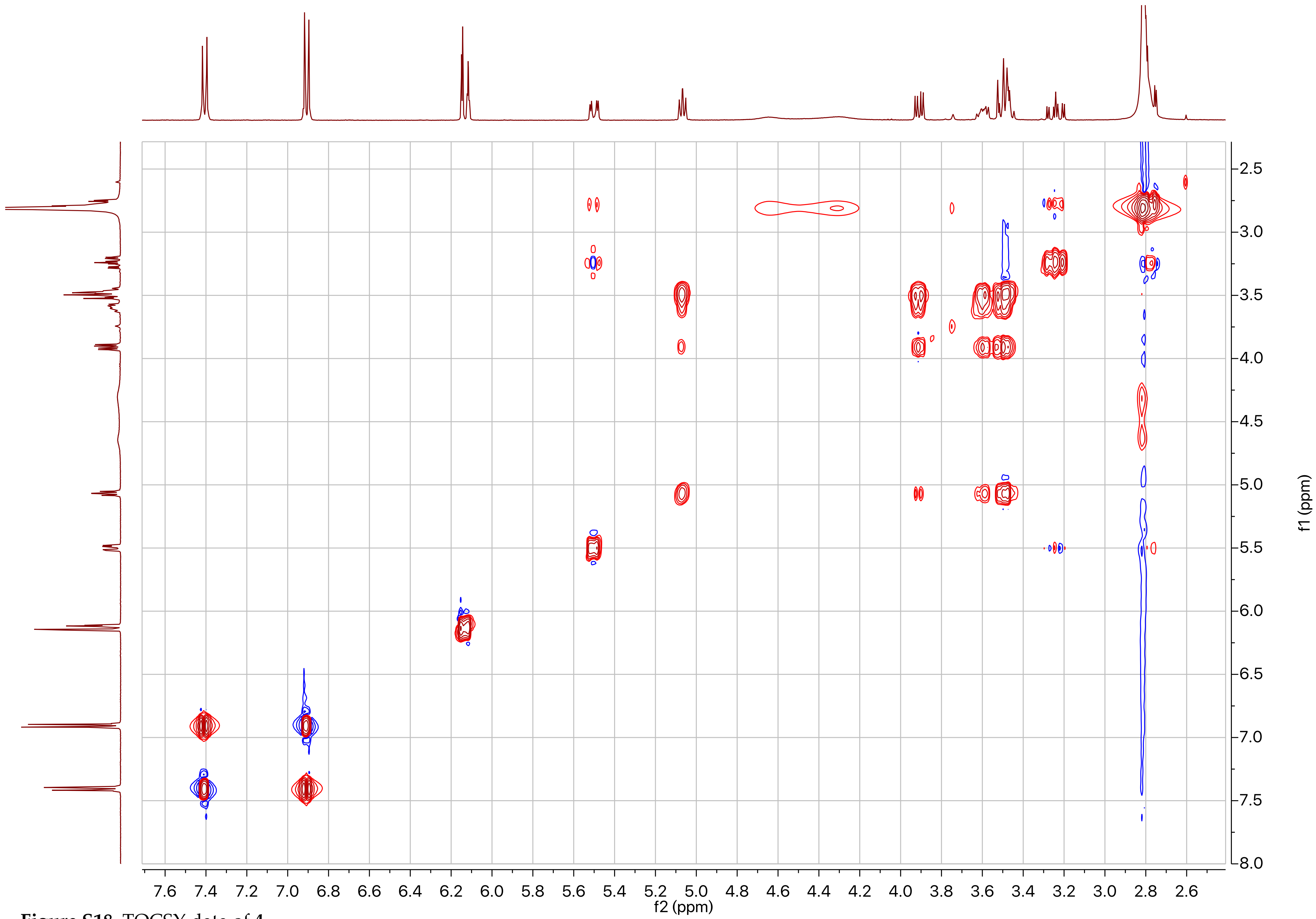

Figure S18. TOCSY data of **4**.

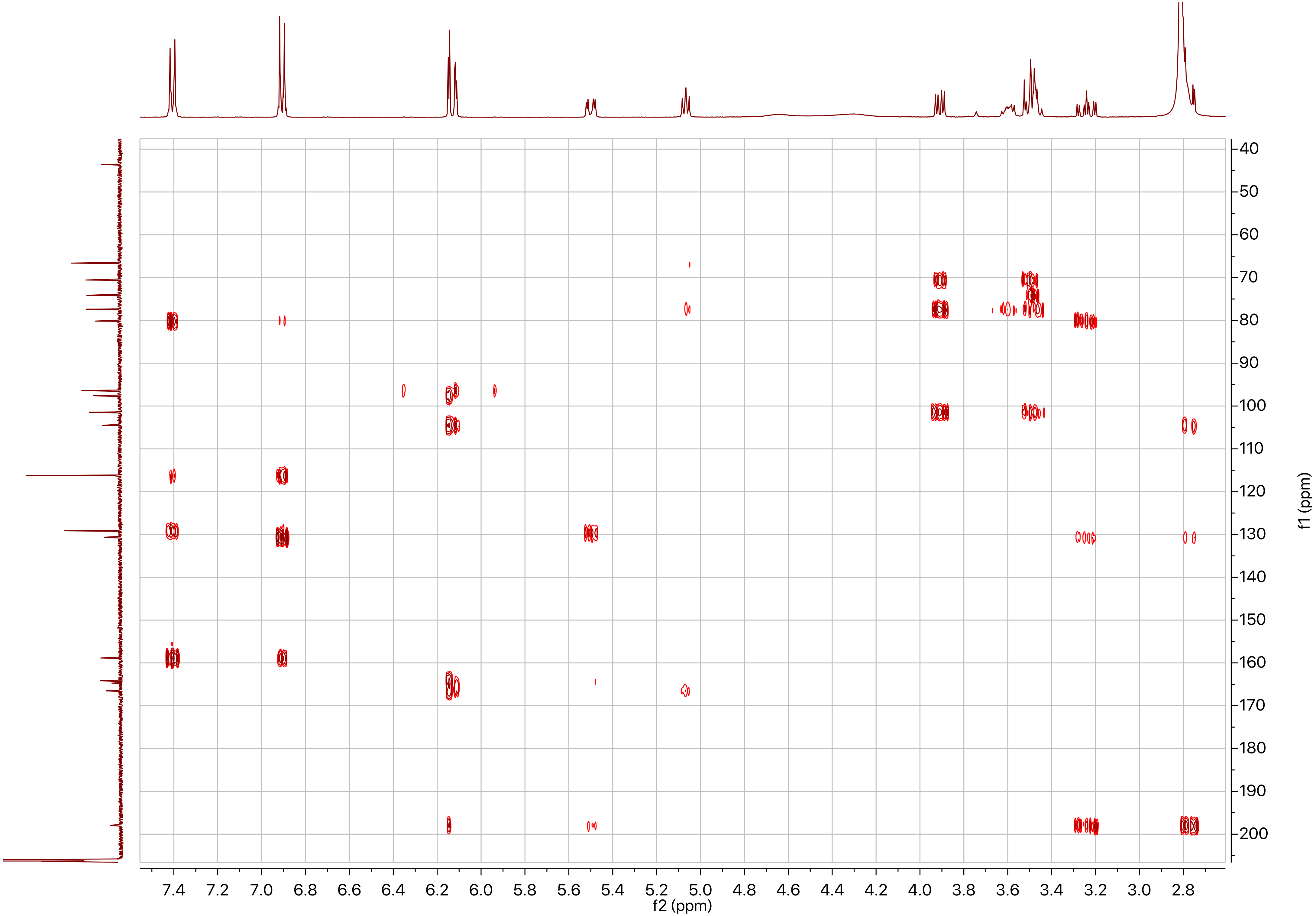

Figure S19. HMBC data of **4**.
